# Hyper-Acetylation of Histone H3K56 Limits Break-Induced Replication by Inhibiting Extensive Repair Synthesis

**DOI:** 10.1371/journal.pgen.1004990

**Published:** 2015-02-23

**Authors:** Jun Che, Stephanie Smith, Yoo Jung Kim, Eun Yong Shim, Kyungjae Myung, Sang Eun Lee

**Affiliations:** 1 Department of Cellular and Structural Biology, University of Texas Health Science Center at San Antonio, San Antonio, Texas, United States of America; 2 Program of Radiation Biology, Department of Radiation Oncology, University of Texas Health Science Center at San Antonio, San Antonio, Texas, United States of America; 3 Genome Instability Section, Genetics and Molecular Biology Branch, National Human Genome Research Institute, National Institutes of Health, Bethesda, Maryland, United States of America; 4 Department of Molecular Medicine, Institute of Biotechnology, Universsity of Texas Health Science Center at San Antonio, San Antonio, Texas, United States of America; Baylor College of Medicine, UNITED STATES

## Abstract

Break-induced replication (BIR) has been implicated in restoring eroded telomeres and collapsed replication forks via single-ended invasion and extensive DNA synthesis on the recipient chromosome. Unlike other recombination subtypes, DNA synthesis in BIR likely relies heavily on mechanisms enabling efficient fork progression such as chromatin modification. Herein we report that deletion of *HST3* and *HST4*, two redundant de-acetylases of histone H3 Lysine 56 (H3K56), inhibits BIR, sensitizes checkpoint deficient cells to deoxyribonucleotide triphosphate pool depletion, and elevates translocation-type gross chromosomal rearrangements (GCR). The basis for deficiency in BIR and gene conversion with long gap synthesis in *hst3*Δ *hst4*Δ cells can be traced to a defect in extensive DNA synthesis. Distinct from other cellular defects associated with deletion of *HST3* and *HST4* including thermo-sensitivity and elevated spontaneous mutagenesis, the BIR defect in *hst3*Δ *hst4*Δ cannot be offset by the deletion of *RAD17* or *MMS22*, but rather by the loss of *RTT109* or *ASF1*, or in combination with the H3K56R mutation, which also restores tolerance to replication stress in *mrc1* mutants. Our studies suggest that acetylation of H3K56 limits extensive repair synthesis and interferes with efficient fork progression in BIR.

## Author Summary

Chromatin poses a barrier to the recombination process. Chromatin modification is therefore a prerequisite factor for the efficient execution of the recombination event. Chromatin remodeling and several unique histone modifications at or near DNA double strand breaks (DSBs) facilitate early recombination processes, but little is known how chromatin state impinges on post-invasion steps of recombination, such as repair synthesis through homologous template, particularly recombination subtypes such as break-induced replication (BIR) involving extensive repair synthesis. Here, we investigated the effect of deletions in chromatin modification and remodeling genes on BIR and discovered that hyper-acetylation of H3K56 selectively impairs BIR and gene conversion associated with long DNA gap synthesis. We also found that hyper-acetylation of H3K56 interferes with the recovery from replication stress in checkpoint deficient cells and induces translocation-type gross chromosomal rearrangements (GCRs). The results provide a basic understanding of how histone modification facilitates efficient fork progression in recombination, controls the types of the repair products and sustains chromosome integrity upon induction of genotoxic stress.

## Introduction

DNA damage drives mutagenesis and chromosomal rearrangements. Homologous recombination (HR) removes DNA lesions primarily at S/G2 phase of the cell cycle by pairing broken DNA ends with intact homologous template and copying across DNA breaks [1]. Break-induced replication (BIR) is the subtype of homologous recombination (HR) that eliminates one-ended DNA breaks or two-ended double strand breaks (DSBs) in the event when only one end of the DNA break is homologous to a template, such as a collapsed replication fork or eroded telomere. By searching for and copying from homologous sequences, often synthesizing hundreds of kilobases of DNA in the process, BIR has been implicated in catalyzing alternative lengthening of telomeres (ALT) and restoring replication forks [2,3]. Extensive DNA synthesis is unique to BIR or to only a subset of gene conversion events, both of which depend on Pol32, a non-essential subunit of DNA polymerase delta [4,5]. The large-scale DNA synthesis during BIR is also highly mutagenic in nature [6]. The initiation step of BIR is frequently associated with template switching, suggesting that the replication fork remains unstable and is prone to dissociation [7]. The precise mechanism behind the high propensity for mutations in BIR is not yet clear but might stem from its conservative mode of DNA synthesis and unique reliance on the helicase Pif1 for bubble migration [8,9,10].

Every DNA transaction including DNA replication and repair occurs within chromatin. Therefore, histone modification and the reconfiguring of chromatin structure are not only a prerequisite, but also dictate both the efficiency and outcome of these events. Temporally disrupted chromatin in DNA replication and repair is then restored by the deposition of new nucleosomes and the re-establishment of unperturbed chromatin architecture globally and locally [11].

Histone H3K56 is an evolutionarily conserved residue that is subjected to reversible acetylation [12,13,14,15,16,17]. Unlike many other modifications at histone tails, H3K56 is located within the globular core domain near the entry and exit sites of nucleosomes, and does not appear to affect DNA-histone interactions as well as chromatin configuration [12,13,18,19]. In *Saccharomyces cerevisiae*, H3K56 is acetylated by the combined action of the acetyltransferase Rtt109/Vps75 and the histone chaperone Asf1, and deacetylated by two redundant class III histone deacetylases (HDACs) Hst3 and Hst4 [16,17,20,21,22]. Functionally, acetylated H3K56 facilitates replication-coupled nucleosome assembly and replication-independent histone exchange [23,24]. H3K56 acetylation is also required for histone eviction and transcription activation at certain promoters [25]. In mammals wherein acetylation of H3K56 is less abundant than in yeast, H3K56 is also methylated by the histone lysine methyltransferase G9a and promotes PCNA docking on chromatin in G1 [26].

In addition to its role in nucleosome assembly during replication and transcription, H3K56 also contributes to DNA repair and signaling [12,27]. Dys-regulation of histone H3K56 acetylation leads to a severe sensitivity to DNA damaging agents and elevated genome instability. Both excess and reduced levels of H3K56 acetylation disrupt cell cycle progression and induce spontaneous DNA damage and hypersensitivity to DNA damaging agent treatment [16,17,20,27,28]. Both hyper- and hypo-acetylation of H3K56 result in defects in sister chromatid cohesion and recombination [29,30] and ribosomal DNA (rDNA) amplification [31], as well as increased mutation frequency and GCR rate [32]. Furthermore, H3K56 is tightly regulated by the cell cycle and DNA damage-induced checkpoint, as the amount of Hst3 fluctuates at both the transcriptional and posttranscriptional level [16,29]. Deletion of *HST3* and *HST4* also leads to thermo-sensitivity, defective telomere silencing and increased chromosome loss [33,34].

Considering the role of H3K56 acetylation in nucleosome assembly, some of these repair defects could be attributed to low nucleosome density and the inability to recover from cell cycle arrest upon DNA damage, particularly in the absence of other histone chaperones [35,36,37]. However, this alone cannot explain why excess acetylation of H3K56 also leads to DNA damage sensitivity and genome instability. It is likely that hyper-acetylation of H3K56 interferes with some other aspect of DNA repair and signaling beyond nucleosome assembly.

In this study, we investigated how chromatin modifiers and remodelers control BIR-mediated repair of an HO endonuclease-induced break on a disomic chromosome. Given the functional overlap of players between BIR and general DNA synthesis where chromatin modifications are tightly coupled to ongoing replication fork progression, we surmise that BIR should also depend on chromatin modification for efficient fork progression. We found that hyper-acetylation of H3K56 specifically inhibits efficient repair synthesis, therefore leading to BIR and gene conversion defects associated with long gap synthesis, but not other HR pathways. Deletion of *HST3* and *HST4* also impairs recovery from a collapsed replication fork in S-phase in DNA damage checkpoint deficient *mrc1*Δ cells and elevates translocation-type GCRs. We propose that acetylation of histone H3 impinges on the post-invasion step in recombination and thereby chromosomal integrity after DNA damage.

## Results

### Deletion of *HST3* and *HST4* leads to BIR deficiency

**Fig 1:**
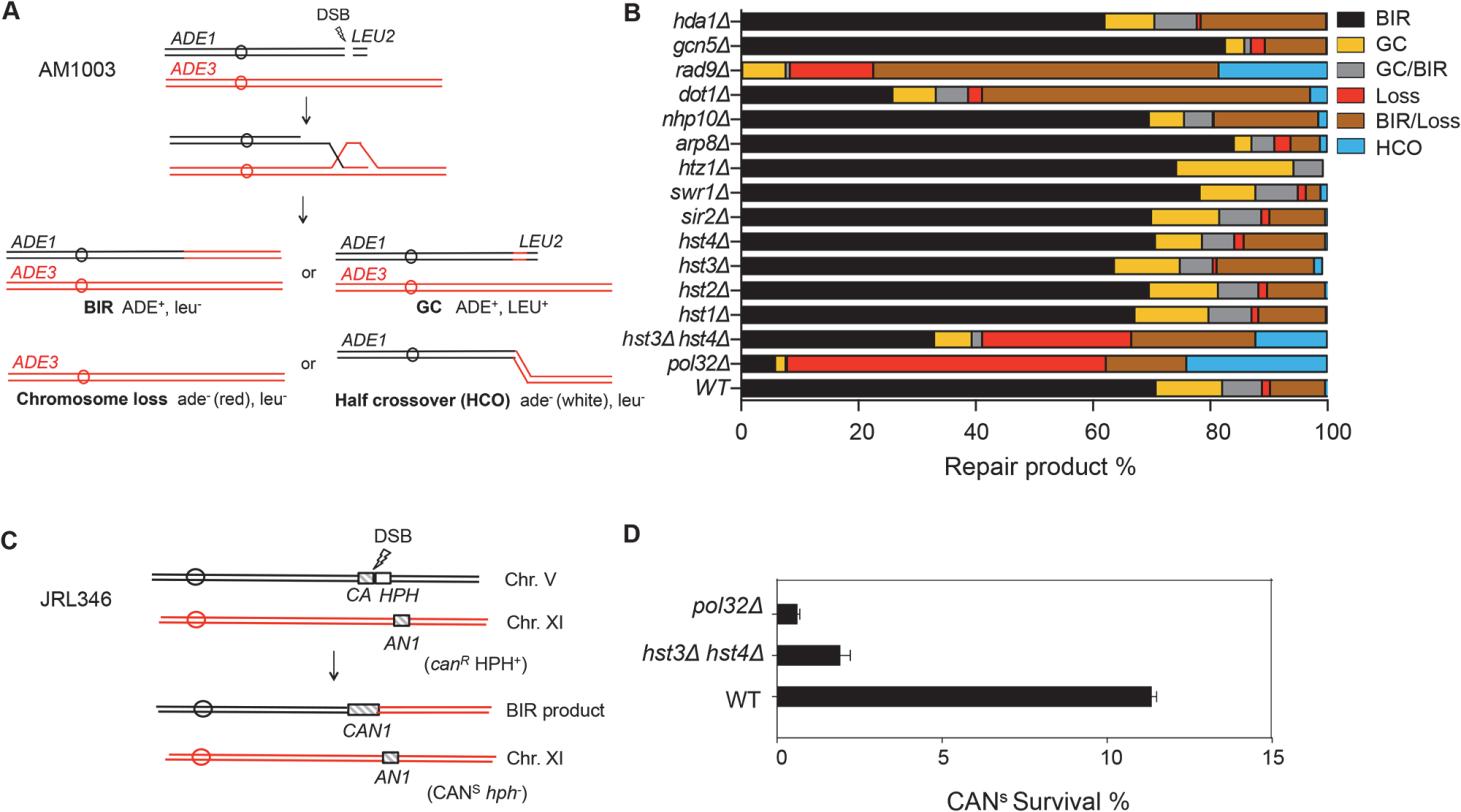
Deletion of *HST3* and *HST4* inhibits BIR. **A**, Schematic view of disomic chromosome III system (AM1003) for analyzing break-induced replication (BIR) in *Saccharomyces cerevisiae*. Four major types of pathways and the corresponding repair products are shown and distinguished by markers (*ADE1*, *ADE3* and *LEU2*) and the sizes on PFGE gels. BIR, break-induced replication; GC, gene conversion; HCO, half crossover. **B**, Deletion of chromatin related factors and their effect on repair outcome in disomic BIR assay. **C**, Schematic view of an ectopic BIR assay. HO recognition site is positioned adjacent to the C-terminally truncated *CAN1* gene (*CA*) on chromosome V and the N-terminally truncated *CAN1* (*AN1*) on Chromosome XI as the homologous template. *HPH*, hygromycin B. **D**, Efficiency of BIR in *pol32*Δ, and *hst3*Δ *hst4*Δ cells, as measured by canavanine sensitive colony formation following DSB induction. Error bars represent s.d.

Extensive DNA synthesis in BIR likely relies on chromatin modification to achieve efficient fork progression. To address this question, we examined the effect of chromatin modifier gene deletions on BIR using an assay measuring BIR between disomic chromosomes [38]. Initial analysis was focused on chromatin remodeling complex factors (Swr1, INO80, RSC) and histone modifiers (Gcn5, Rtt109, Hst3, Hst4, and Dot1) implicated in DNA DSB repair. We induced a DSB at the *MAT* **a** locus on a truncated copy of chromosome III, thereby triggering centromere proximal DNA ends to invade and copy from the intact *MATα-inc* allele on a full-length chromosome III as a DSB repair template (Fig. 1A). Since the truncated chromosome III possesses limited homology (46 bp) to the *MATα-inc* allele on the telomere proximal side of the DSB, BIR is the primary repair option, although a lower frequency of other repair events such as gene conversion (GC), chromosome loss or half crossover still occurs. The small percentage (>10%) of sectored Ade+/− colony formation likely represents the cases where only one of the two sister chromatids completed repair. Different types of repair events were deduced by the presence of marker genes located on the left arm of chromosome III (*ADE1* and *ADE3*) and the *LEU2* gene of the truncated chromosome III in surviving colonies. The survival frequency is nearly 100% because cells still retain a functional chromosome III copy even if repair is aborted and the truncated chromosome is lost.

Inactivation of most chromatin modification factors altered the repair profile and BIR frequency but to a moderate extent (Fig. 1B). Increased BIR frequency in several of these mutants (*htz1*, *arp8*, and *swr1*) could be explained by their reduced resection function because extensive resection is known to suppress BIR [39,40]. However, the effect of *SWR1* or *ARP8* deletion on resection is variable among studies and modest at best [41,42,43]. We also found that deletion of *DOT1* led to a dramatic decrease in BIR but increase in BIR/chromosome loss events. Since Dot1 recruits the checkpoint adaptor protein Rad9 at DNA break sites and plays an important role in DNA damage checkpoint activation [44], BIR deficiency in *dot1*Δ cells can be attributed to the lack of a functional cell cycle checkpoint. Indeed, *dot1*Δ and *rad9*Δ mutants showed very similar repair profiles with elevated BIR/chromosome loss (sectoring) events. Alternatively, BIR defects in *dot1*Δ or *rad9*Δ might be due to the increased resection in these mutants reported earlier [45].

Interestingly, deletion of two class III HDACs, *HST3* and *HST4* reduced BIR frequency but increased chromosome loss and half crossover events. The result somewhat mirrored that of *pol32*Δ mutants, albeit with less severity. Deletion of *HST3* or *HST4* alone or other sirtuin genes including *SIR2* did not reduce BIR frequency. We also monitored BIR product formation by pulsed-field gel electrophoresis (PFGE) of genomic DNA isolated at various time points post-HO expression and Southern blot hybridization with a probe that anneals to the *ADE1* gene on chromosome III. We found that deletion of *HST3* and *HST4* led to a 2-fold reduction in BIR product formation by Southern blot assay (S1 Fig.). To further confirm whether *hst3*Δ *hst4*Δ mutant cells are defective in BIR, we performed another ectopic BIR assay, wherein a galactose-inducible HO endonuclease creates a DSB on the left end of chromosome V and BIR restores an intact *CAN1* gene that renders the cells canavanine sensitive, but leads to the loss of the *HPH* gene distal to the DSB (Fig. 1C). Using this ectopic assay we found that deletion of *HST3* and *HST4* led to a 5-fold reduction in BIR (Fig. 1D). These results indicate that *hst3*Δ *hst4*Δ mutants are significantly deficient in BIR.

The strong resemblance in repair profiles between *hst3*Δ *hst4*Δ and *pol32*Δ mutants prompted us to examine if *hst3*Δ *hst4*Δ cells exhibit reduced expression of Pol32. We assessed the amount of triple HA-tagged Pol32 in *hst3*Δ *hst4*Δ cells via immunoblot assay with anti-HA antibody. We found that deletion of *HST3* and *HST4* did not reduce the expression level of Pol32 (S2 Fig.).

### Hyper-acetylation of H3K56 inhibits break-induced replication

**Fig 2:**
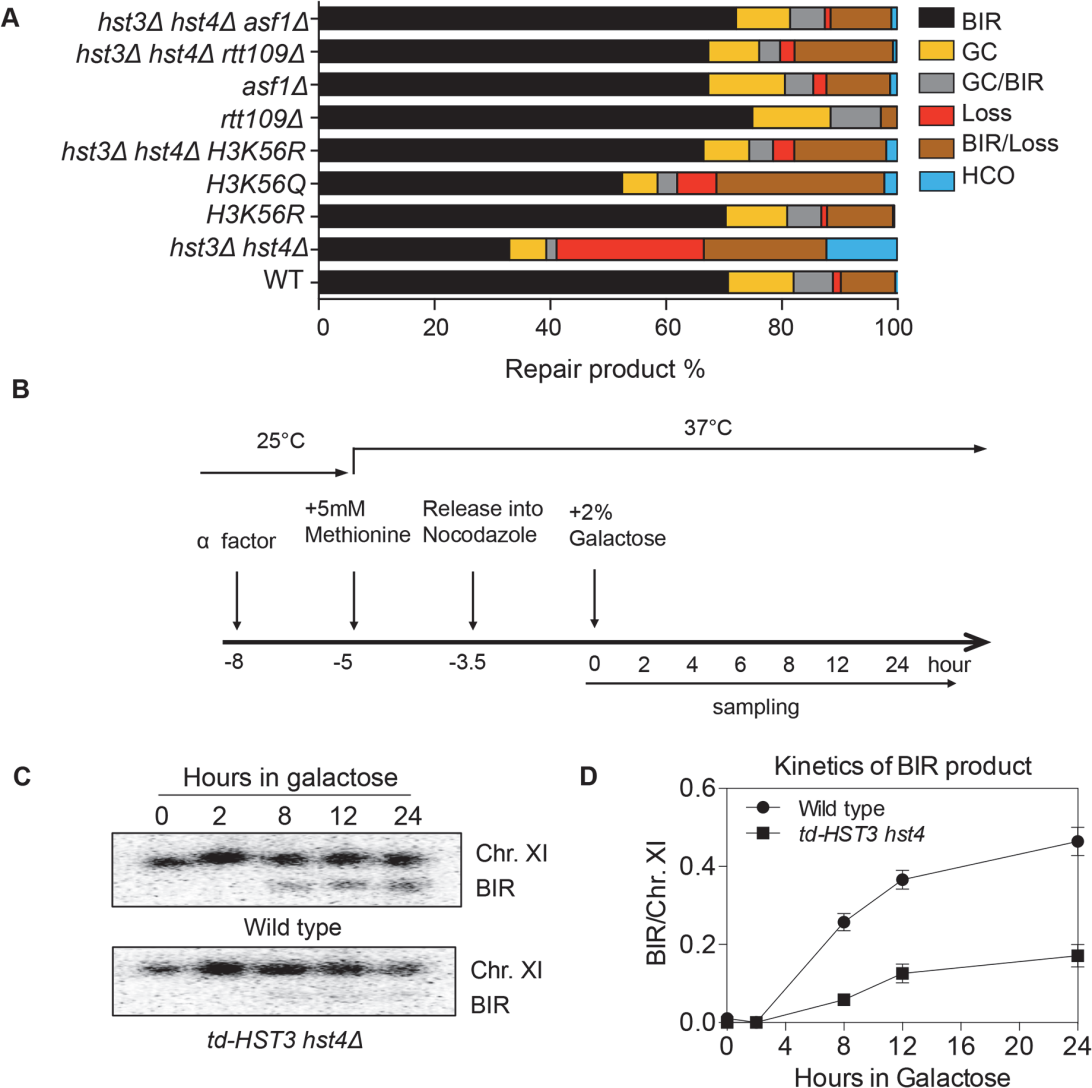
Hyper-acetylation of H3K56 causes BIR defects. **A**, Various combination of mutants as shown were tested in the disomic BIR system. Shown are the averages of at least two independent experiments. BIR, break-induced replication; GC, gene conversion; HCO, half crossover. An average of 500–1000 events were scored for each experiment. **B**, Flowchart showing experimental procedure. **C**, Southern blot analysis of BIR product formation in cells depleted of Hst3. Chromosomes were separated by PFGE and a DNA probe specific for *MCH2* was used to detect BIR products. **D**, Quantification of Southern blot results.

Histone H3K56 is the best-known target of Hst3/Hst4 and represents the marker of newly deposited nucleosomes during replication or repair [12,17]. We therefore tested whether H3K56R or H3K56Q mutations that either block or partially mimic H3K56 acetylation will alter BIR frequency. We found that the H3K56R mutation alone did not exert any effect on BIR efficiency, whereas H3K56Q led to a slight but reproducible BIR defect (Fig. 2A). The modest BIR deficiency in H3K56Q mutants compared to *hst3*Δ *hst4*Δ cells supports the notion that H3K56Q does not fully mimic acetylated H3K56 [12]. We then analyzed the effect of the H3K56R mutation on BIR frequency in *hst3*Δ *hst4*Δ. If the observed BIR deficiency in *hst3*Δ *hst4*Δ is due to hyper-acetylation of H3K56, then the K56R substitution should offset this BIR defect. Indeed, H3K56R almost fully rescued the BIR defect in *hst3*Δ *hst4*Δ cells (Fig. 2A). These results suggest that hyper-acetylated H3K56 inhibits BIR.

Rtt109 is an acetyltransferase responsible for acetylation of H3K56 [20,21]. Asf1 is a histone chaperone specific for H3K56 acetylation [22]. We thus examined the effect of *RTT109* or *ASF1* deletion on BIR frequency in wild-type and *hst3*Δ *hst4*Δ cells using the disomic BIR assay. As predicted, *rtt109*Δ or *asf1*Δ alone did not influence BIR frequency (Fig. 2A). However, deletion of *ASF1* or *RTT109* in *hst3*Δ *hst4*Δ mutants improved BIR frequency to a level almost identical to that in wild-type cells. The level of BIR product formation observed in this genetic assay was also confirmed by Southern blot analysis (S1 Fig.). These results further demonstrate that hyper-acetylation of H3K56 inhibits BIR.

### Hyper-acetylation of H3K56 does not inhibit other recombination subtypes

BIR deficiency in *hst3*Δ *hst4*Δ cells prompted us to test whether deletion of *HST3* and *HST4* generally affects other HR processes. We thus tested the effect of *HST3* and *HST4* deletion on mating-type switch recombination, ectopic gene conversion, and single strand annealing using genetic assays wherein the repair of an HO break occurs by different recombination subtypes (S3 Fig.). The recombination frequency was determined by survival frequency after HO expression and physical monitoring of the repair products by Southern blot-based assay. We found that none of the recombination subtypes tested were impaired in *hst3*Δ *hst4*Δ cells (S3 Fig.). We also determined the frequency of non-homologous end joining (NHEJ) in donorless *hst3*Δ *hst4*Δ cells by inducing an HO break at the *MAT* locus. Deletion of *HST3* and *HST4* did not lead to any changes in survival frequency following HO expression (S3 Fig.). This suggests that *hst3*Δ *hst4*Δ mutants are deficient in BIR but not other recombination or NHEJ processes.

Since H3K56 acetylation marks newly assembled nucleosomes, half of the nucleosomes in each cell should bear this mark after DNA synthesis. Based on this premise, we set out to test if the physiologically relevant level of H3K56 acetylation is sufficient to inhibit BIR. To this end, we engineered a yeast strain expressing a degron-fused and the MYC epitope-tagged Hst3 as the sole H3K56 deacetylase, and then examined BIR efficiency in cells arrested at G2 after one round of DNA synthesis upon depletion of Hst3 using the ectopic BIR assay (Fig. 2B). Depletion of Hst3 at non-permissive temperature (37°C) was confirmed by thermosensitivity and immunoblot assay with anti-MYC antibody (S4A and S4C Fig.). Cell cycle progression was monitored by fluorescence activated cell sorting (FACS) after propidium iodide (PI) staining of cells (S4B Fig.). We found that depletion of Hst3 decreased BIR frequency 3-fold in cells arrested at G2 as compared to those expressing Hst3 (Fig. 2C, 2D). The relatively small reduction (3-fold) in BIR in Hst3-depleted cells compared to the five-fold reduction observed in *hst3 hst4* deleted cells suggests that the level of BIR is proportional to the amount of intracellular H3K56 acetylation (S4D Fig.). Inactivation of Hst3 and Hst4 by the pan–sirtuin inhibitor nicotinamide (NAM) also reduced BIR frequency 2-fold (S5 Fig.). As shown previously, deletion of *SIR2*, *HST1* and *HST2* did not alter BIR frequency (Fig. 2A). The results suggest that physiological levels of H3K56 acetylation are sufficient to suppress BIR in cells.

### Hyper-acetylation of H3K56 suppresses recovery from replication stress in checkpoint deficient *mrc1* cells

Replication forks stall when deoxyribonucleotide triphosphate (dNTP) pools are depleted and even collapse without a functional S-phase checkpoint [46]. Mrc1 is an S-phase checkpoint protein that stabilizes replication forks when hydroxyurea (HU) is used to induce dNTP pool depletion [47,48]. In the absence of Mrc1, stalled replication forks harbor extensive single strand DNA (ssDNA) and become collapsed [49]. Thus cell survival relies on pathways that repair collapsed replication forks [50]. We examined HU sensitivity of *mrc1*Δ cells deleted for *HST3*, *HST4* or *POL32* genes upon acute HU treatment. Unlike typical plate based assays where cells are exposed chronic DNA damage and dys-regulation of H3K56 acetylation leads to HU sensitivity [28], the acute HU exposure (1 to 4 h) employed here likely addresses the repair of collapsed fork [51].

**Fig 3:**
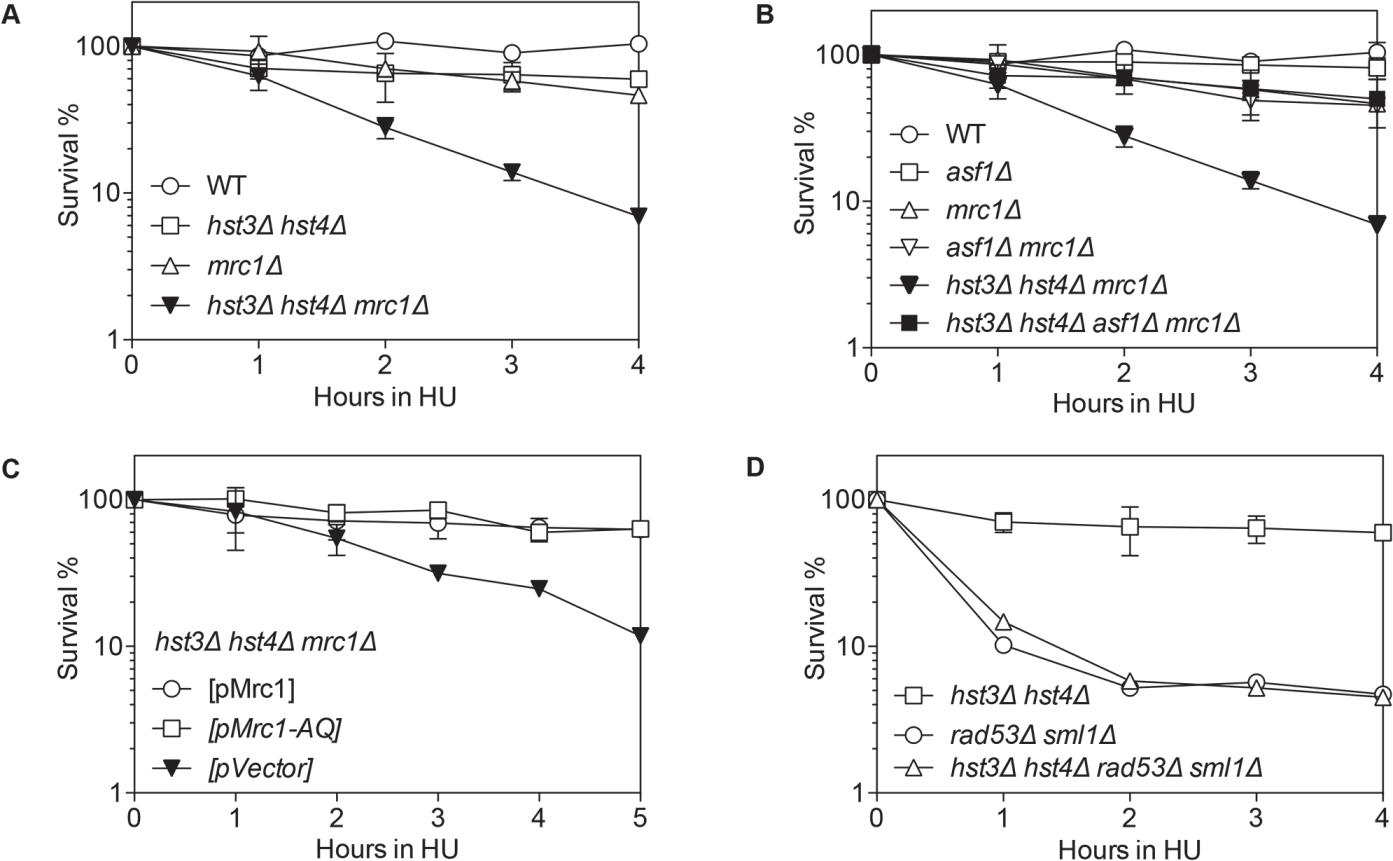
Synergistic HU sensitivity between *hst3*Δ *hst4*Δ and *mrc1*Δ but not *rad53*Δ. Cells with indicated genotypes grown in log phase were alpha factor arrested for 3 hours and released into YEPD or SC-Ura with 150 mM HU up to 5 hours. Percent survival of *hst3*Δ *hst4*Δ, *mrc1*Δ, *hst3*Δ *hst4*Δ *mrc1*Δ (A), *asf1*Δ, *mrc1*Δ, *asf1*Δ *mrc* Δ, *asf1*Δ *hst3*Δ *hst4*Δ *mrc1*Δ (B), *hst3*Δ *hst4*Δ *mrc1*Δ supplemented with the plasmids expressing Mrc1 or mrc1AQ (C), *hst3*Δ *hst4*Δ, *rad53*Δ and *hst3*Δ *hst4*Δ *rad53*Δ (D) are shown. Percent survival was calculated by the number of colonies treated with HU divided by the number of colonies before HU treatment and determined at every hour after release from α factor arrest. Plotted are the mean values of three independent experiments ± s.d.

We found that *hst3*Δ *hst4*Δ, *pol32*Δ or *mrc1*Δ cells are only mildly sensitive to HU treatment, but *hst3*Δ *hst4*Δ *mrc1*Δ or *pol32*Δ *mrc1*Δ cells showed far greater sensitivity than each single gene deletion mutant (Figs. 3A and S6A Fig.). Deletion of *POL32* also sensitizes *tof1*- or *csm3*-deleted cells to HU treatment (S6B–S6C Fig.). Furthermore, the HU sensitivity of *hst3*Δ *hst4*Δ *mrc1*Δ mutants is fully rescued by an *asf1*Δ mutation (Fig. 3B). Deletion of *ASF1* or *RTT109* did not sensitize *mrc1*Δ to HU treatment (Figs. 3B and S6D). These results suggest that the observed HU-induced stall or collapse of replication forks in *mrc1*Δ cells relies upon Pol32- and H3K56 deacetylation dependent process for recovery.

Rad53 is another S-phase checkpoint protein that sustains replication fork integrity in response to HU treatment [46]. Surprisingly, in contrast to *mrc1*Δ cells, deletion of *HST3* and *HST4* or *POL32* did not further sensitize *rad53*Δ mutants to HU treatment Fig. 3D). These results suggest that not all collapsed forks depend on Hst3/Hst4 and Pol32-dependent process for repair.

Mrc1 contributes to both damage-induced checkpoint induction and replication fork stabilization [47]. These two functions can be uncoupled by the *mrc1AQ* allele that abolishes Mrc1’s checkpoint function but retains its role in replication fork stabilization [47]. To determine which function of Mrc1 contributes to HU resistance in *hst3*Δ *hst4*Δ or *pol32*Δ mutants, we examined HU sensitivity in a strain bearing the *mrc1AQ* mutation and *hst3 hst4* or *pol32* gene deletions. We found that the *mrc1AQ* mutation did not confer synergistic HU sensitivity to *hst3*Δ *hst4*Δ or *pol32*Δ cells, suggesting that Mrc1’s checkpoint function is dispensable for HU sensitivity in *hst3*Δ *hst4*Δ or *pol32*Δ mutants (Figs. 3C and S6E). Additionally, deletion of *RAD9*, another DNA damage checkpoint adaptor but not a fork stabilizer, did not sensitize *hst3*Δ *hst4*Δ cells to HU treatment (S6F Fig.). The results suggest that stalled/collapsed replication forks but not checkpoint dysfunction lead to HU sensitivity in *hst3*Δ *hst4*Δ or *pol32*Δ mutants.

**Fig 4:**
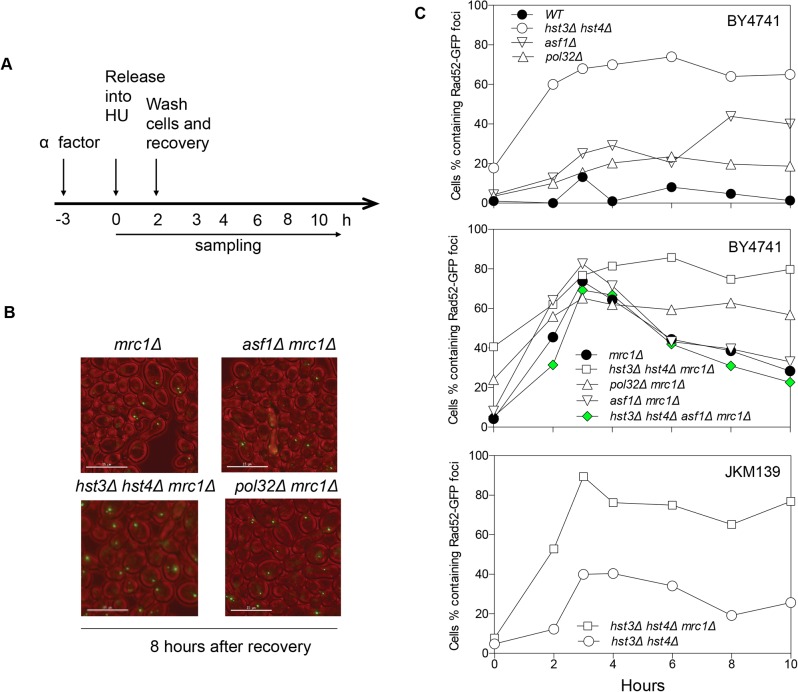
Rad52-GFP foci in *hst3*Δ *hst4*Δ *mrc1*Δ and *pol32*Δ *mrc1*Δ cells persist after HU treatment. **A**, Flowchart showing experimental procedure. **B**, Representative images for different cells showing persistent Rad52-GFP foci at 8 hours after recovery from HU treatment. Size bar, 15 µm. **C**, Quantification of percentage of Rad52-GFP foci in the cell population. At least 300 individual cells were counted for each genotype and time point.

The lethality upon acute HU treatment in *hst3*Δ *hst4*Δ *mrc1*∆ cells could be due to an inability to repair a collapsed fork or alternatively to a more extensive fork collapse. It has been shown that HU treatment induces Rad52-green fluorescent protein (GFP) focus formation in *mrc1*Δ but not in wild-type nuclei, likely marking the site of stressed and collapsed replication forks and their repair [51]. We thus monitored the kinetics of Rad52-GFP foci as a surrogate for the repair of collapsed replication forks in order to dissect HU-induced fork collapse in *mrc1*∆ cells. We found that Rad52-GFP foci appeared in *mrc1*Δ cells upon HU treatment, reached a maximum level after releasing cells into fresh YEPD medium, and gradually disappeared most likely due to repair (Fig. 4). Importantly, in *hst3*Δ *hst4*Δ *mrc1*Δ cells and *pol32*Δ *mrc1*Δ cells, the Rad52-GFP foci persisted for up to 8 h after HU treatment, suggesting that *hst3*Δ *hst4*Δ or *pol32*Δ cells are deficient in repairing collapsed replication forks (Fig. 4). To test if the repair deficiency in *hst3*Δ *hst4*Δ *mrc1*Δ mutants could be attributed to hyper-acetylation of H3K56, we deleted *ASF1* and monitored Rad52-GFP kinetics following HU treatment. Deletion of *ASF1* led to a high level of Rad52-GFP foci at 6–8 h after release from HU, consistent with the role of Asf1 in fork stability [52], yet significantly offset the level of Rad52-GFP in *hst3*Δ *hst4*Δ *mrc1*Δ cells (Fig. 4A and B). Deletion of *HST3* and *HST4* also led to an increase in spontaneous Rad52-GFP focus formation and sensitivity to acute HU treatment in the BY4741 strain, but not in another genetic background (JKM139, see Fig. 4A and C, and S6 Fig.). We surmise that Hst3 and Hst4 contribute to the integrity of replication forks in certain genetic backgrounds. The results support the role of Hst3, Hst4 and Pol32 in the repair of collapsed replication forks.

### Hyper-acetylation of H3K56 increases gross chromosomal rearrangements

Improper repair of replication stress is a likely source of gross chromosomal rearrangements (GCRs) [53]. Furthermore, deletion of *ASF1* or *RTT109* increases GCR rate [20,54]. We thus analyzed GCR frequency in *hst3*Δ *hst4*Δ or *pol32*Δ cells using the assay that measures the loss of GCR reporter genes on the left arm of chromosome V (Table 1). We found that deletion of *HST3* and *HST4* increased GCRs 457-fold (Table 1). Deletion of *ASF1* partially reduced GCR rate to the level indistinguishable from that observed in *asf1*Δ cells, suggesting that hyper-acetylation of H3K56 is the likely cause of elevated GCRs in *hst3*Δ *hst4*Δ mutants. Interestingly, deletion of *POL32* leads to only a mild increase in GCR. Analysis of the breakpoints of GCRs showed that most of the GCR events in *hst3*Δ *hst4*Δ cells are DNA translocations with 3–12 bp of microhomology at the junction, whereas all GCRs in *pol32*Δ cells are the result of *de novo* telomere formation (Tables 1 and S1).

**Table 1:** Gross Chromosomal Rearrangement (GCR).

Strain	GCR Rate (Fold)	Telomere Addition Rate (Fold)	Translocation Rate (Fold)
Wild type^1^	3.5 X 10^–10^ (1)	2.9 X 10^–10^ (1)	5.8 X 10^–11^ (1)
*hst3Δ hst4Δ*	1.6 X 10^–7^ (457)	5.8 X 10^–8^ (200)	1.0 X 10^–7^ (1745)
*asf1Δ*	2.5 X 10^–8^ (71)	2.0 X 10^–8^ (70)	4.5 X 10^–9^ (78)
*pol32Δ*	1.0 X 10^–8^ (28)	1.0 X 10–^8^ (34)	0
*hst3Δ hst4Δ asf1Δ*	3.2 X 10^–8^ (91)	1.5 X 10^–8^ (52)	1.7 X 10^–8^ (286)

1. Data from Myung et al. [46]

### BIR defect in *hst3Δ hst4Δ* is distinct from thermosensitivity and DNA damage sensitivity

**Fig 5:**
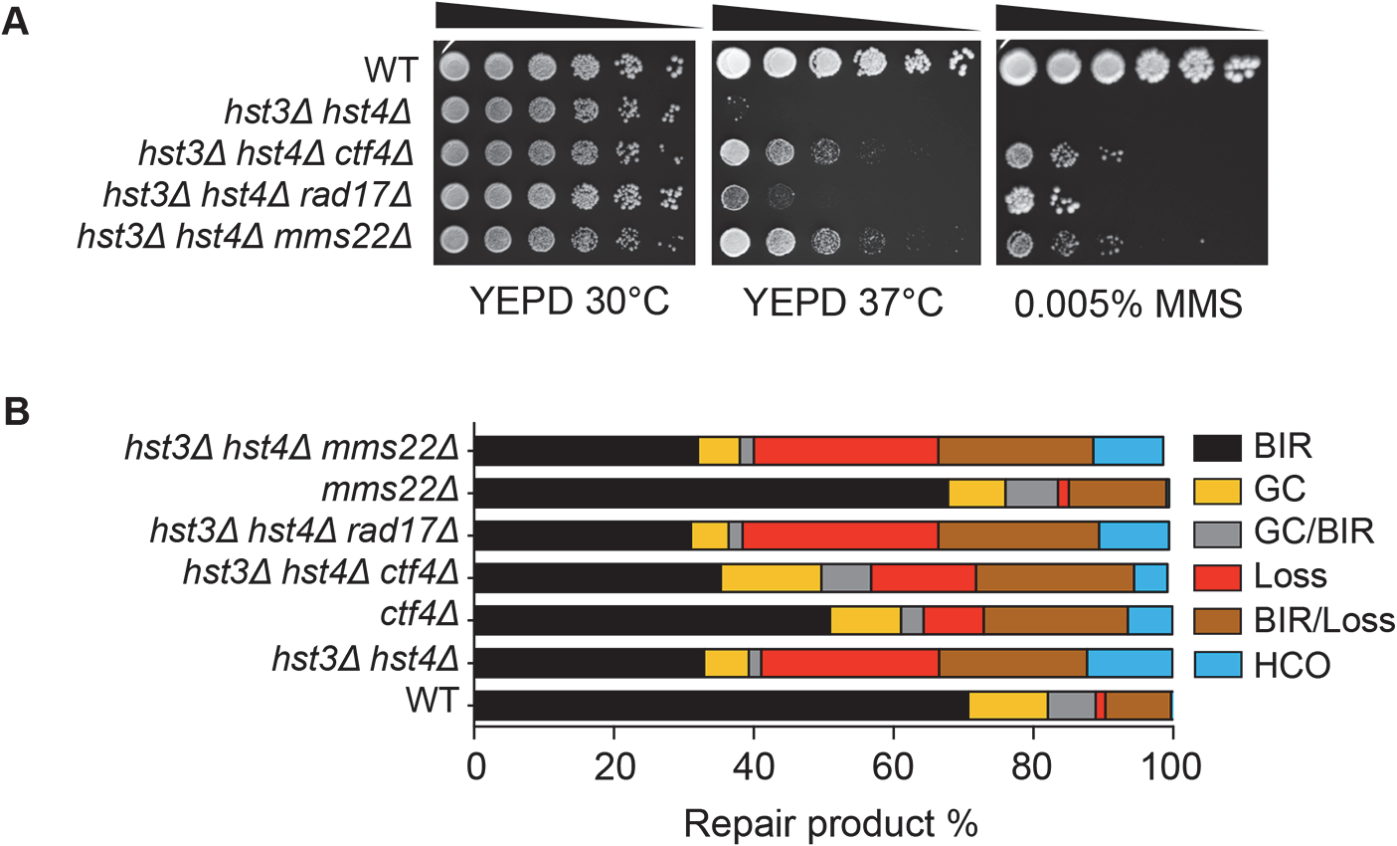
Deletion of genes improving thermo- and MMS resistance of *hst3*Δ *hst4*Δ cells does not rescue BIR defects. **A**, Serial dilutions of cells with indicated genotypes were spotted and cultured at 30°C or 37°C with or without 0.005% MMS. Pictures were taken after 4 days. **B**, Repair outcomes of wild-type (WT) and indicated mutant cells. Shown are the averages of at least two independent experiments. BIR, break-induced replication; GC, gene conversion; HCO, half crossover. An average of 500–1000 events were scored for each experiment.

Cells bearing the *hst3*Δ *hst4*Δ mutations do not grow at 37°C but the deletion of genes involved in DNA damage checkpoint induction (*RAD17*) or sister chromatid establishment (*CTF4*) partially offset their thermosensitivity and DNA damage sensitivity phenotypes [28]. To test if the observed thermosensitivity and BIR defect share a common genetic cause, we deleted *RAD17* or *CTF4* and examined whether the gene deletions improve BIR in *hst3*Δ *hst4*Δ cells. We found that deletion of *RAD17* or *CTF4* partially offsets the growth deficiency at 37°C and resistance to MMS treatment as predicted, but does not affect defects in BIR in *hst3*Δ *hst4*Δ mutants (Fig. 5A and B). The results suggest that thermosensitivity, DNA damage sensitivity, and BIR deficiency are not genetically linked to each other in *hst3*Δ *hst4*Δ mutants and likely represent somewhat distinct molecular defects.

Most recently, a ubiquitin ligase complex containing Rtt101, Mms1, and Mms22 has been implicated in H3K56 acetylation-dependent mutation avoidance and damage tolerance pathways [32]. Deletion of *MMS22* also offsets the thermosensitivity of *hst3*Δ *hst4*Δ cells (Fig. 5A and [55]). We thus tested the effect of *MMS22* gene deletion on BIR using the disomic BIR assay. We found that deletion of *MMS22* did not alter the repair profile in wild-type or *hst3*Δ *hst4*Δ cells (Fig. 5B). The results further support the model that the BIR defect in *hst3*Δ *hst4*Δ mutants is genetically distinct from underlying molecular defects causing thermosensitivity, elevated mutagenesis and sensitivity to DNA damaging agents.

Evidence suggests that acetylated H3K56 promotes SWR-C dependent exchange of H2AZ to H2A at promoter proximal regions and thereby regulates transcription [56]. If the observed BIR defect in *hst3*Δ *hst4*Δ mutants is due to excessive exchange of H2AZ to H2A, deletion of *HTZ1* should lead to BIR defects as in *hst3*Δ *hst4*Δ cells. However, we found that deletion of *HTZ1* or *SWR1* does not result in BIR deficiency in the disomic BIR assay (Fig. 1B). Furthermore, deletion of *HTZ1* or *SWR1* did not rescue the BIR defect in *hst3*Δ *hst4*Δ cells (S7 Fig.). The results suggest that BIR deficiency in *hst3*Δ *hst4*Δ mutants is not due to excessive removal of H2AZ or dynamic histone exchange at promoters.

### Hyper-acetylation of H3K56 inhibits repair synthesis

We then asked how hyper-acetylation of H3K56 inhibits BIR, which involves a few common and unique recombination steps [2]. Following DSB induction, the broken DNA ends are processed by 5’ to 3’ resection to yield single stranded DNA (ssDNA) that serves as the binding site for Rad51 recombinase. The ssDNA-Rad51 complex (a.k.a. pre-synaptic filament) then invades into a homologous template and initiates strand pairing. The 3’ non-homologous tail should be removed from the annealed DNA so that repair synthesis will begin copying from the template until the end of the chromosome. We analyzed the formation of ssDNA, binding of Rad51 at donor and recipient DNA molecules, 3’ flap removal, and initiation of DNA synthesis in *hst3*Δ *hst4*Δ mutant cells using PCR-based assays and chromatin immunoprecipitation (ChIP) using anti-Rad51 antibody (S8, S9, S10 Figs.).

**Fig 6:**
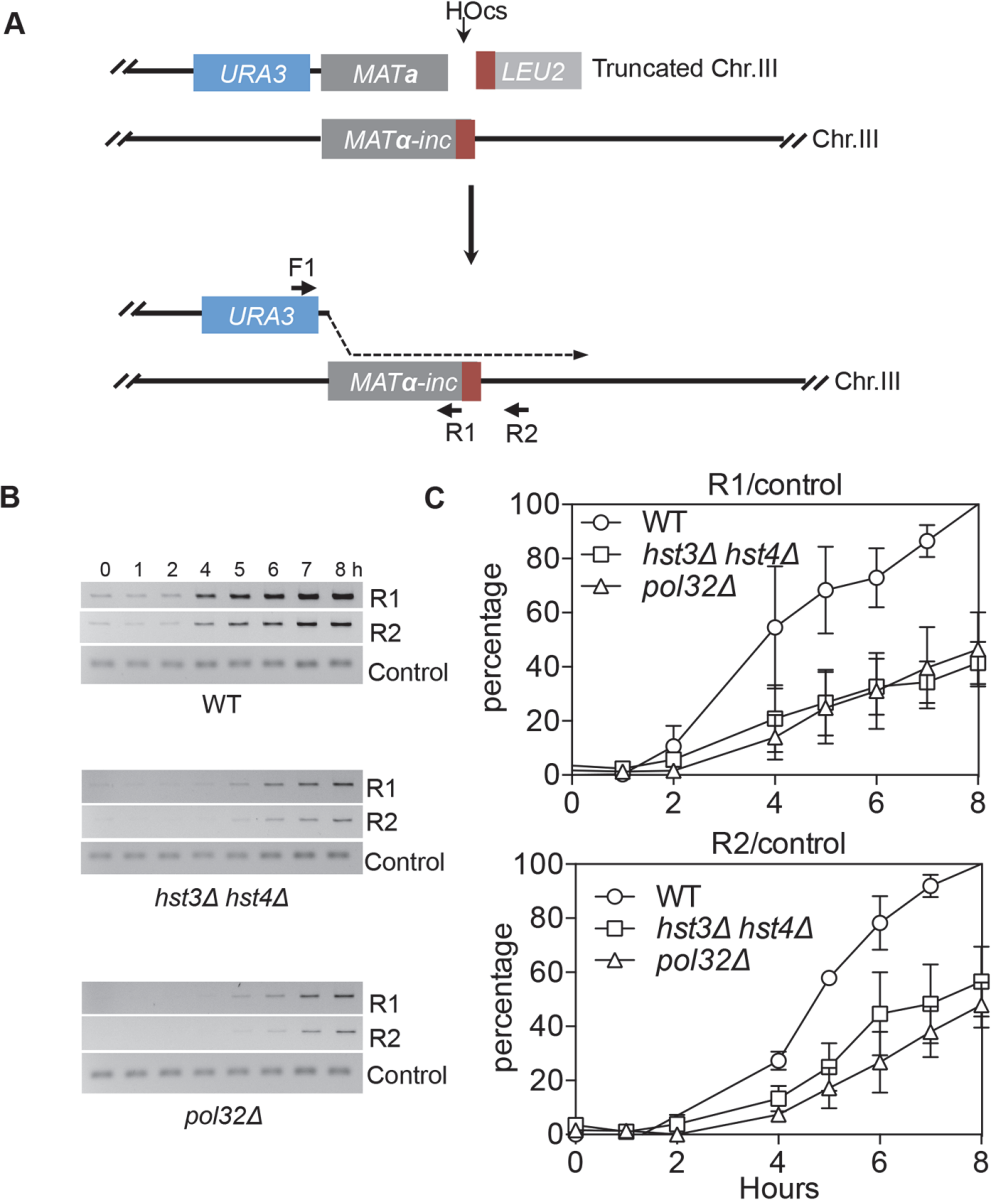
*hst3*Δ *hst4*Δ and *pol32*Δ mutants are defective in the DNA synthesis step of BIR. **A**, BIR synthesis was determined by semi-quantitative PCR using upstream PCR primer (F1) annealing to the *URA3* gene inserted upstream of the *MAT*a gene and downstream primers annealing either to a *MAT*α-inc specific sequence (R1) or downstream (R2). **B**, Representative DNA agarose gel images of semi-quantitative PCR. **C**, Quantification of the gel images. Plotted are the means of percent repair products at indicated time points post-HO expression from three independent experiments ± s.d.

**Fig 7:**
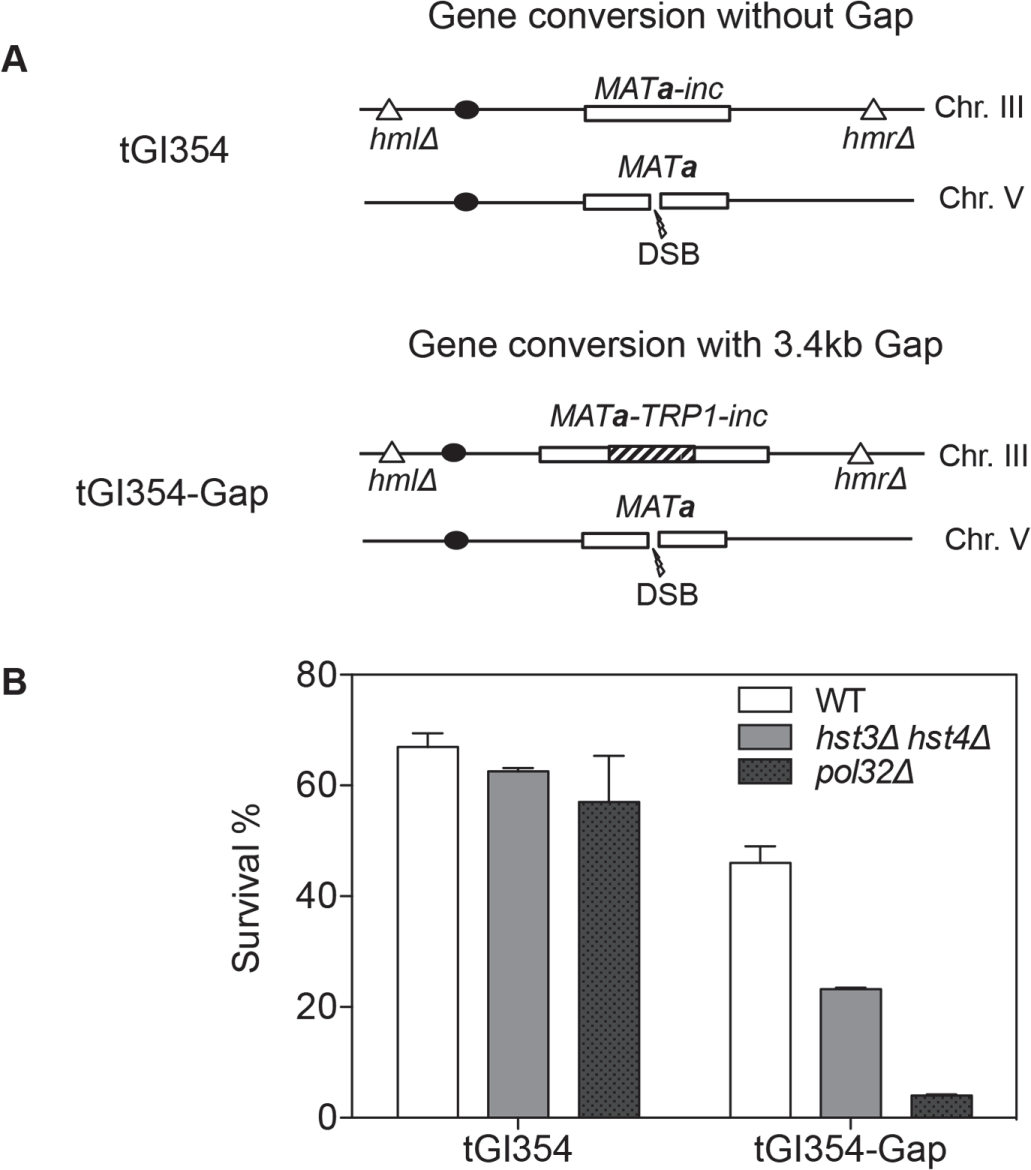
*hst3*Δ *hst4*Δ and *pol32*Δ mutants are defective in DNA synthesis during gene conversion with a 3.4-kb gap. **A**, The *TRP1* gene was inserted into *MAT* **a**-inc on chromosome III in tGI354 to generate tGI354-gap strain. **B**, Percent survival in isogenic strains with indicated genotypes and/or a 3.4-kb gap. Plotted are the mean values of three independent experiments ± s.d.

We found that deletion of *HST3 HST4* did not impair resection or the kinetics of Rad51 enrichment at either the break or homologous donor sites (see S8, S9 Figs. for experimental details). We discovered a slight reduction in flap removal efficiency in both *hst3*Δ *hst4*Δ and *pol32*Δ cells, but such a mild deficiency does not likely account for the BIR defects observed in these mutants (S10 Fig.). Furthermore, *hst3*Δ *hst4*Δ and *pol32*Δ mutants are not defective in single strand annealing wherein 3’ flap removal becomes the rate-limiting step, dictating repair frequency (S3 Fig.). We next examined the kinetics of initial repair synthesis by PCR amplification of repair products using a pair of primers in which one anneals to the donor site and the other anneals to the recipient DNA molecule. We discovered that both *hst3*Δ *hst4*Δ and *pol32*Δ mutants were severely defective in repair synthesis (Fig. 6). A single round of DNA replication after depletion of Hst3 in *hst4* Δ cells also inhibits repair synthesis during BIR (S11 Fig.). In contrast, deletion of *HST3* and *HST4*, or *POL32* did not significantly impair or alter the kinetics of repair synthesis in mating type switching consistent with the finding that mating type switching is proficient in these mutants (S3 Fig.). The results suggest that the BIR defect in *hst3*Δ *hst4*Δ cells might be due to inefficient DNA synthesis. The results also reinforce that two-end break repair with no or limited gap is fundamentally different from two-end break repair with large gap or one-end break repair [57]. To further test if *hst3*Δ *hst4*Δ mutants are deficient in long repair DNA synthesis, we determined the frequency of gene conversion that requires 3.4-kb gap synthesis in wild-type, *hst3*Δ *hst4*Δ, and *pol32*Δ cells (Fig. 7A). As evident in Fig. 7B, deletion of *HST3* and *HST4* significantly reduces gene conversion requiring large gap synthesis (tGI354-Gap), although *pol32*Δ cells exhibited a more severe defect (Fig. 7B). This is consistent with the relatively greater deficiency of *pol32*Δ mutants in BIR and repair synthesis (see Fig. 1B). Neither *hst3*Δ *hst4*Δ nor *pol32*Δ exhibited reduced gene conversion frequency without a gap (tGI354). Cumulatively, our data support the model that hyper-acetylation of H3K56 inhibits extensive repair synthesis.

## Discussion

Chromatin modification is an integral part of DNA replication and repair. By searching for chromatin modifiers involved in BIR, we discovered that deletion of two redundant sirtuins *HST3* and *HST4* impairs extensive repair synthesis to suppress BIR and gene conversion with long gap synthesis. We also demonstrated that Hst3/Hst4 and Pol32 facilitate recovery from collapsed replication forks when either of the S-phase checkpoint proteins *MRC1* or *TOF1* is deleted. Deletion of *hst3 hst4* also led to an increase in translocation-type GCRs. These results suggest that histone modification modulates BIR and chromosomal integrity.

### Hyper-acetylation of histone H3K56 inhibits BIR

BIR entails extensive DNA synthesis even to the end of the chromosome. Since chromatin may impede processive DNA synthesis [11], we surmise that BIR likely depends on chromatin modification, which then enables extensive DNA synthesis to the chromosome end. By surveying several candidate genes that have already been implicated in DNA synthesis and recombination, we discovered multiple chromatin modifiers that impinge on BIR events, supporting our original premise that chromatin modification represents an integral part of BIR. Interestingly, the effect of deleting several chromatin modifier genes on BIR can be explained by their role in resection, highlighting the impact of resection regulation on the frequency of BIR events [39,40,58]. The results also indicate that chromatin regulation comprises a major component in early repair steps, often contributing to the commitment stage of DNA repair. Consistent with this, chromatin configuration has been implicated in repair pathway channeling in several model systems [59,60].

Our results also led to the discovery that deletion of other histone modifiers, specifically *HST3* and *HST4*, renders cells severely deficient in BIR using two different BIR assays, with a repair profile closely resembling that of *pol32*Δ mutants. Several observations indicated that hyper-acetylation of H3K56 causes BIR deficiency in *hst3*Δ *hst4*Δ cells. First, the H3K56Q mutation that mimics persistent acetylation reduced the frequency of BIR, albeit more mildly than *hst3*Δ *hst4*Δ. Second, deletion of *ASF1* or *RTT109*, or replacement of lysine 56 with arginine in a histone H3 mutation that blocks H3K56 acetylation rescued the BIR defect in *hst3*Δ *hst4*Δ mutants. Third, *hst3*Δ *hst4*Δ cells are sensitive to HU in the presence of a collapsed replication fork, but become resistant to HU when *ASF1* is deleted. We also showed that the intracellular level of H3K56 is proportional to BIR frequency and that a physiologically relevant level of H3K56 acetylation is sufficient to repress BIR. Lastly, deletion of *HST3 HST4* elevated the frequency of translocation-type GCRs, but this increase was partially suppressed by the concomitant deletion of *ASF1*. Together these results suggest that H3K56 status, but no other *HST3* and *HST4* targets, is responsible for BIR deficiency and the associated chromosomal instability.

To define how H3K56 acetylation inhibits BIR, we assessed the integrity of the four major recombination steps in BIR: resection, Rad51 binding at donor and recipient DNA molecules, 3’ flap removal and repair synthesis. Our findings suggest that deletion of *HST3* and *HST4* specifically impairs repair synthesis. This helps explain why only BIR and gene conversion via long gap repair synthesis, but no other recombination pathways, are deficient in *hst3*Δ *hst4*Δ cells. The results also support our premise that epigenetic regulation represents a key component of BIR.

How does hyper-acetylation of H3K56 impede repair synthesis? It should be possible that acetylation of H3K56 might compromise the establishment of a stable replication fork at a BIR template. Interestingly, results from chromatin immunoprecipitation (ChIP) assays demonstrated that enrichment of Pol delta is reduced in *hst3*Δ *hst4*Δ cells, whereas Pol alpha and Pif1 still associate at the replication fork with almost equal efficiency (S12 Fig.). However, reduced association of Pol delta at a BIR template could simply be the effect but not the cause for BIR deficiency in *hst3*Δ *hst4*Δ mutants. Alternatively, acetylation might block replication fork movement by conferring nucleosomes inherently resistant to sliding or evicting from the template strand. Indeed, deletion of *HST3* and *HST4* in one genetic background (BY4741) led to an increase in spontaneous Rad52-GFP focus formation and elevated sensitivity to HU treatment. However, structural and biochemical analysis of histone octamers carrying H3K56Q did not reveal any major change in nucleosome architecture [18]. Furthermore, hyper-acetylation of H3K56 might impair access to or the activities of topoisomerase I or helicases, each of which relieves helical torsions or catalyzes strand unwinding ahead of a replication fork. In either case, we surmise that frequent fork stalling may ensue in *hst3*Δ *hst4*Δ mutants due to non-processive DNA synthesis, which may underlie the increased spontaneous DNA lesions and mutagenesis exhibited by these cells. The observations that many phenotypes of *hst3*Δ *hst4*Δ cells can be suppressed at least partially by improving replication processivity support this model [28]. Further studies are needed to determine precisely how H3K56 acetylation inhibits repair synthesis.

In mitotic cells, limiting extensive repair synthesis in recombination should be beneficial as it could reduce mutagenesis inherent to repair synthesis [61]. The size of gene conversion track length also positively correlates with the frequency of crossovers and the rate of loss of heterozygosity [62]. Limiting the amount of repair synthesis should also helps avoid unequal sister chromatid exchange event and sustain repeat stability as seen in rDNA or triplet repeats. Interestingly, DNA damage induces proteolytic degradation of Hst3 [16,29], preserving intracellular level of H3K56 acetylation, which could then help limit excessive repair synthesis during recombinational repair of DNA damage.

### Hyper-acetylation of H3K56 interferes with recovery from collapsed forks

Since collapsed replication forks produce one-ended DSB, BIR has been implicated in the restart of replication upon fork collapse [2,3]. Nevertheless, multiple means are still available in cells to complete full genome duplication without re-establishing forks by BIR: a) use of a replication fork approaching from a neighboring origin; b) template switching or re-priming. Accordingly, the precise role of BIR in recovery from a collapsed fork has not yet been fully defined.

Interestingly, we discovered that deletion of *POL32* or *HST3* and *HST4* caused a severe sensitivity to HU when *MRC1* is also deleted. Synergism between *MRC1* and *HST3/HST4* in resistance to HU treatment can be attributed to deficient maintenance of fork integrity in *mrc1* mutants but not to an S-phase checkpoint defect, since the checkpoint-deficient *mrc1AQ* allele fully rescued HU sensitivity. Puzzlingly however, in *rad53*Δ, where a majority of replication forks collapse, deletion of *POL32* or *HST3* and *HST4* did not sensitize cells to HU treatment. Evidence suggests that the structure of a collapsed fork in *mrc1* mutants upon HU treatment associates with extensive ssDNA formation (2–3 kb long) due to uncoupling of the replicative helicase from stalled fork [49]. Extensive ssDNA at the fork then leads to DNA breakage and the repair requires extensive repair synthesis through newly deposited, H3K56 acetylated nucleosomes on the donor DNA molecule (S13 Fig.). The unique architecture associated with stalled forks in *mrc1* cells might also be ill-suited for re-priming or bypass-mediated recovery, and thereby relies on the role of BIR like process as a bona fide replication restart mechanism. Alternatively, hyper-HU sensitivity in *hst3*Δ *hst4*Δ *mrc1*Δ mutant could be attributed to the excessive fork collapse due to a deficiency in stalled fork protection under HU treatment. However, we did not detect massive fork collapses in *hst3*Δ *hst4*Δ *mrc1*Δ or *mrc1*Δ cells as compared to *rad53*Δ using the assays that monitor the frequency of Rad52-GFP foci or DNA fragmentation by PFGE (Figs. 4 and S14).

The typical collapsed replication forks in cells do not likely feature long ssDNA and such forks in principle contain acetylated H3K56 only at or behind the fork. This raises a possibility that the effect of persistent H3K56 acetylation shown in this study is not applicable to general collapsed fork recovery, but only to those with forks collapsed with long ssDNA as seen in *mrc1*- or *tof1*-deleted cells. Furthermore, H3K56 acetylation is increased in multiple types of cancers [14] and the results shown here could be more relevant to those pathological conditions or at regions of chromatin with more persistent H3K56 acetylation.

### Deletion of *HST3* and *HST4* leads to GCR

Previous studies suggest that both hypo- and hyper-acetylation of histone H3K56 elevates GCR events [20,32,54]. Consistent with this, we also detected dramatically increased GCRs in *hst3*Δ *hst4*Δ mutants. However, unlike in an *asf1* deletion [54], most GCR events in *hst3*Δ *hst4*Δ cells are chromosomal translocations. The results indicate that the molecular defects and pathways leading to GCR events in these strains are distinctly different. Accordingly, deletion of *ASF1* partially decreased the GCR rate in *hst3*Δ *hst4*Δ cells to a level comparable to that in *asf1*Δ cells.

We have evidence to suggest that it is unlikely that increased GCR observed in *hst3*Δ *hst4*Δ mutants is derived from a BIR defect. If BIR deficiency alone is sufficient for elevated GCR, deletion of *POL32* that results in a more severe deficiency in BIR should increase GCR events at a greater extent than in *hst3*Δ *hst4*Δ cells. Instead, we found that deletion of *POL32* only slightly increases GCRs. Analysis of the types of GCR and the breakpoint junctions in these two mutants further demonstrated that the BIR defect in *hst3*Δ *hst4*Δ cells cannot account for its elevated GCR because *pol32*Δ produces *de novo* telomere addition but not chromosomal translocations as seen in *hst3*Δ *hst4*Δ cells. Why then is GCR increased in *hst3*Δ *hst4*Δ mutants? One possibility is that DNA replication forks are unstable in *hst3*Δ *hst4*Δ cells and lead to frequent stall and collapse during S-phase. Spontaneous Rad52-GFP, DDC2-GFP, and Mre11-GFP focus formation, as well as Rad53 phosphorylation supports the increase in replication-associated DNA damage in *hst3*Δ *hst4*Δ cells, which then probably fuels the formation of GCRs. In fact, many cellular phenotypes of *hst3*Δ *hst4*Δ mutants can be explained by fork instability that might lead to such pleiotropic phenotypes. The idea is further supported by the observation that multiple phenotypes associated with *hst3*Δ *hst4*Δ mutants including thermosensitivity, drug sensitivity and spontaneous mutagenesis can be partially offset by *RAD17*, *ELG1*, or *MMS22* deletion that catalyzes the S-phase checkpoint, PCNA unloading or replication fork stability, respectively. However, these mutations did not offset BIR deficiency, indicating that the basis of the observed BIR defect is distinctly different from that of other phenotypes.

Analysis of GCR breakpoint junctions shows that most GCR events in *hst3*Δ *hst4*Δ cells are chromosomal translocations with microhomology (MH) at the breakpoint junctions. The presence of MH suggests that GCR events in *hst3*Δ *hst4*Δ mutants proceed by microhomology-mediated (MM)-BIR or microhomology-mediated end joining (MMEJ) mechanisms. Notably, deletion of *POL32* not only impairs BIR but also the MMEJ pathway [5,63]. Therefore, lack of GCRs in *pol32*-deleted cells may be due to their inability to carry out MMEJ. Indeed, all the GCR events in *pol32* are *de novo* telomere formation. We also found that *hst3*Δ *hst4*Δ mutants are not deficient in MMEJ. Alternatively, it is possible that the GCR events derived from residual BIR mechanisms in *hst3*Δ *hst4*Δ cells and the severe BIR deficiency in *pol32*Δ cells disable the formation of translocation-type GCRs.

In all, our study has revealed the role of H3K56 acetylation in inhibiting extensive repair synthesis and restarting of stressed replication forks in checkpoint-deficient cells. It will be interesting to test if acetylation of H3K56 also inhibits collapsed fork recovery and BIR in human cells. It is noteworthy that HDAC or sirtuin inhibitors have been widely regarded as promising cancer therapeutic agents. Our study has implications for how these inhibitors target checkpoint-defective tumors cells and highlight their utility to treat cancer cells in combination with replication stress agents.

## Materials and Methods

### Strains

Disomic BIR strains are derivatives of AM1003 with genotype *MAT* **a**-*LEU2-*tel/*MAT*α-*inc ade1 met13 ura3 leu2-3*,*112/leu2 thr4 lys5 hml*Δ::*ADE1/hml*Δ::*ADE3 hmr*Δ::*HYG ade3*::*Gal-HO* FS2Δ::*NAT/FS2* [38]. Ectopic BIR strains are derivatives of JRL346 [40]. HU sensitivity was tested on derivatives of JKM139 with genotype *ho*Δ *MAT* **a** *hml*Δ::*ADE1 hmr*Δ::*ADE1 ade1-100 leu2-3*,*112 lys5 trp1*::*hisG’ ura3-52 ade3*::*GAL*::*HO* [64]. JKM161, YMV80, tGI354 were used for mating-type switch, SSA, and ectopic recombination assays, respectively [65,66]. Gene conversion with a 3.4-kb gap strain was constructed by insertion of a *TRP1* containing DNA fragment originating from the pFA6a-*TRP1* plasmid into the *MAT* **a**-inc template of tGI354. The yeast strain expressing MYC epitope tags and degron fusion was constructed by PCR amplification of degron cassette as described in [17,67]. The strain list is shown in S2 Table.

### BIR assay

The assay was performed as previously described with minor changes in cell culture[38]. BIR derivative strains were cultured in SC-Ade medium overnight and then transferred to YEP-glycerol medium and cultured for more than 6 h before plating on YEP-galactose plates. Cells cultured in this way are selected against spontaneous chromosome loss. An aliquot of cells was also plated on YEPD plates. After colonies grew up on the plates for 3–5 days, they were replica-plated onto SC-Leu and SC-Ade plates to determine their genotypes. At least 500 individual colonies were scored and at least 2 different isolates with the same genotype were analyzed. Colonies replica-plated from YEPD plates were also examined to determine the background chromosome loss. BIR frequency of ectopic BIR assay was determined as described in [5].

To physically monitor BIR products, 3×10^7^ cells were harvested to make DNA plugs for each time point. Pulsed-field gel electrophoresis (PFGE) was run in a 1.2% agarose gel with switch time 10–35s, voltage 6v/cm, angle 120°, 14°C for 32–40 h (disomic BIR assay) or with switch time 50–70 s, voltage 6v/cm, angle 120°, 14°C for 21–24 h (ectopic BIR assay). PCR products from *ADE1* gene or *MCH2* fragment were radiolabeled and used to detect BIR bands by Southern blot assay.

To test BIR in Hst3-depleted cells or those treated with NAM, *HST4*-deleted cells were arrested in G1 by treating them with 10 µg/ml alpha-factor, subjected to incubation at 37°C (non-permissive conditions) for an additional 1.5 h, releasing cells into nocodazole containing (15 µg/ml) media for 2.5 h and inducing HO endonuclease by addition of 2% (w/v) galactose. Aliquots of cells were harvested at each indicated time point and subjected to Western blot to investigate the level of Hst3, H3K56 acetylation using anti-MYC (Sigma) and H3K56 acetylation antibodies (Upstate) and FACS analysis for cell cycle progression. Cells were harvested to prepare DNA plugs for PFGE to analyze BIR products and PCR analysis of repair synthesis. PCR products from *ADE1* gene or *MCH2* fragment were radiolabeled and used to detect BIR bands or initial repair products by Southern blot.

### HU sensitivity test

Logarithmically growing cells were alpha-factor arrested for 3 h and released into YEPD medium containing 150 mM HU. Cells were harvested at each time point, washed twice with water and plated onto YEPD plates. The survival rate was calculated by the number of colonies treated with HU divided by the number of colonies before HU treatment. For the *mrc1AQ* experiment, Uracil dropout medium instead of YEPD medium was used for cell culture.

### Microscopy

Images were taken with a DeltaVision microscopy system (Applied Precision). Each original image contained 12–15 Z-stacks. After deconvolution, images were subjected to quick-projection. The projected pictures were then counted for Rad52-GFP foci using SoftwoRx.

### Measurement of 3’ flap cleavage efficiency during gene conversion

3’ flap cleavage efficiency during gene conversion was determined by measuring the retention frequency of yeast centromeric plasmids pFP120 and pFP122 after HO induced DSB as described in [68]. The percentage of plasmid retention is calculated as the fraction of colonies retaining the repaired plasmid on SC–Ura divided by the total number of colonies on YEPD.

### ChIP assay

Chromatin immunoprecipitation (ChIP) assays were performed as described previously [69]. Briefly, JKM161 cell cultures grown to a density between 1 × 10^7^ and 2 × 10^7^ cells/ml in pre-induction medium (YEP-glycerol) were induced for HO endonuclease by adding 2% galactose. For immunoprecipitation, the sonicated extracts were incubated with Rad51 antibody (kindly provided by Dr. Patrick Sung P). Quantitative PCR was performed using *MAT* **a** (break) specific primers and *HML* (donor) specific primers.

### Quantitative PCR-based resection assay

JKM139 derivative cells grown to a density between 1 × 10^7^ and 2 × 10^7^ cells/ml in pre-induction medium (YEP-glycerol) were induced for HO endonuclease by adding 2% galactose. Five ml of cells at different time points were harvested and processed for genomic DNA isolation using Masterpure yeast genomic DNA purification kit (Epicentre). DNA equal to 0.4 ml of cells was digested by 20 units of *Bsa*J1 enzyme or mock digested without *Bsa*J1 for 2 h in a 50 μl reaction volume. Samples were then diluted 10 times and subject to quantitative PCR to determine the percentage of resection. The principle of this assay is shown in S8 Fig. The rate of single stranded DNA formation (r) is calculated by r = (RS_*Bsa*J1 digested_/ RS_Mock digested_)/(Input_*Bsa*J1 digested_/ Input_Mock digested_), where RS indicates PCR value at the *Bsa*J1 restriction site flanking the HO break and Input indicates PCR value at a site from a different chromosome and does not contain the *Bsa*J1 restriction site. Since r = X/(X+2(1-X)) where X is the rate of resection, the percentage of resection was calculated by: 100X% = 200r/(r+1). Primer sequences for determining resection at 3.3 kb, 12.6 kb or input are 5’TCCGAACCAATGTCGTCATCCATAGTATC and 5’GGCGCCTTTAATTCATGGTCTTTACCTTT; 5’TTCTACGGCGACTGGTGGAATTGC and 5’ATGTAGCTTGGCTCTTGCTCAAATGC; 5’ACTTAGTTCTGGAGATTATCGCCTTATCG and 5’CTGTCTTTCGCTTAGTTCACCTCTACC, respectively.

### Analysis of initial DNA synthesis in BIR

Semi-quantitative PCR was performed with Iproof polymerase (Biorad) with a 2 minute extension time and 20 cycles (allelic BIR assay) or 28 cycles (ectopic BIR assay) using Forward primer F (5’ TACTGGAGTTAGTTGAAGCATTAGG3’) upstream of the HO break inside the *URA3* gene and Reverse primer R1 (5’ TGACTTCCAGACGCTATCCTGTGAA 3’) inside the *MAT* locus at an alpha-specific sequence or R2 (5’GATATTAAGTCCTCCGTCCAATCTG3’) downstream of *MAT*alpha-inc, inside the *TAF2* gene (allelic BIR assay) or Forward primer F2 (5’AATATTGTGTGTATGGGCACAAACCCTTG3’) inside the *NPR2* gene and Reverse primer R3 (5’ CCTCAATGTCTCTTCTATCGGAAT3’) that anneals to the carboxy terminus of *CAN1* gene (ectopic BIR assay). Control PCR was performed with 1 minute extension time for 20 cycles using Forward primer F3 (5’TCCATGCTAGATTAGCACACAGTAA3’) and reverse primer R4 (5’CTTTTGTAGGTGTCCTTAATTTCCA3’). PCR products were resolved in a 1% agarose gel and stained with ethidium bromide. The gel pictures were captured by GEL-LOGIC system and PCR bands were quantified by Carestream software. Inversed images were showed in the Fig. 6 and S11 Fig.

### GCR assay

A fluctuation-based test was performed to determine gross chromosomal rearrangements [70]. In brief, 3–5 ml of 11 confluent cultures in YEPD from single colonies were plated on SC medium lacking arginine and supplemented with 60 mg/L L-canavanine and 1 g/L 5-FOA for scoring GCRs. Cells were also diluted and plated on YEPD plates for counting total number of cells. GCR plates were cultured for 5–10 days at 30°C and colony numbers counted. Calculation of GCR rate and determination of junction type (*de novo* telomere formation or translocation) were as previously described [70].

## Supporting Information

S1 Fig Analysis of BIR kinetics in mutants. **A**, Southern blot analysis of BIR product formation. Chromosomes were separated by PFGE and a DNA probe specific for *ADE1* was used to detect BIR products. **B**, Quantification of Southern blot results.
(TIF)

S2 Fig Analysis of Pol32 expression in wild-type and *hst3*Δ *hst4*Δ cells. Cycling cells with Pol32-3xHA were harvested and Western blotting was performed to determine the level of Pol32 expression in *hst3*Δ *hst4*Δ cells. Samples were 1:2 serially diluted for better resolution. Coomassie blue stained PVDF membrane is shown as a loading control.
(TIF)

S3 Fig *hst3*Δ *hst4*Δ cells are not defective in HR or NHEJ. **Left:** Schematic diagram of genetic assays analyzing the integrity of mating-type switching (A), ectopic recombination (B), single strand annealing (C) and non-homologous end joining (D). **Right:** Efficiency of repair in *hst3*Δ *hst4*Δ as measured by viability following a DSB or 120 Gray ionizing radiation (IR) (E). Error bars represent s.d.
(TIF)

S4 Fig Depletion of Hst3 leads to BIR defects. **A**, Serial dilutions of *hst4*Δ cells with or without degron fused (td)-Hst3 were spotted and cultured at 28°C or 37°C. Pictures were taken after 4 days. **B**, Cell cycle progression monitored by fluorescence activated cell sorting (FACS). **C**, The level of Hst3 expression in cells growing at non-permissive (37°C) temperature. Coomassie blue stained PVDF membrane is shown as a loading control. **D**, The level of H3K56 acetylation upon degron-induced depletion of Hst3. The level of H3 is shown as a loading control.
(TIF)

S5 Fig Inhibition of sirtuins causes BIR defects. **A**, Flowchart showing experimental procedure. **B**, Southern blot analysis of BIR product formation in cells treated with 50 mM NAM. Chromosomes were separated by PFGE and a DNA probe specific for *MCH2* was used to detect BIR products. **C**, Quantification of Southern blot results.
(TIF)

S6 Fig HU sensitivity. Percent survival after 150 mM HU for up to 5 h was determined in cells with indicated genotypes: **A**, *pol32*Δ, *mrc1*Δ, *pol32*Δ *mrc1*Δ; **B**, *pol32*Δ, *tof1*Δ and *pol32*Δ *tof1*Δ; **C**, *pol32*Δ, *csm3*Δ, and *pol32*Δ *csm3*Δ; **D**, *rtt109*Δ, *mrc1*Δ, and *rtt109*Δ *mrc1*Δ; **E**, *pol32*Δ *mrc1*Δ supplemented with the plasmids expressing Mrc1 or mrc1AQ, **F**, *hst3*Δ *hst4*Δ, *rad9*Δ and *hst3*Δ *hst4*Δ *rad9*Δ. Plotted are the mean values of three independent experiments ± s.d.
(TIF)

S7 Fig Deletion of *HTZ1 or SWR1* does not suppress BIR defects in *hst3*Δ *hst4*Δ cells. Efficiency of BIR in *hst3*Δ *hst4*Δ, *hst3*Δ *hst4*Δ *htz1*Δ, and *hst3*Δ *hst4*Δ *swr1*Δ cells, as measured by canavanine sensitive colony formation following a DSB using the ectopic BIR assay.
(TIF)

S8 Fig *hst3*Δ *hst4*Δ cells are not defective in resection at the *MAT* locus. **A**, Schematic diagram illustrating the principle of determining DNA resection by quantitative PCR. When DNA is not resected (not resected), DNA can be digested by restriction enzyme (*Bsa*JI, Bs). PCR primers surrounding the restriction enzyme site (grey arrows) cannot yield a PCR product. Upon resection to generate single stranded DNA, the restriction site is lost, and PCR primers surrounding the restriction site yield a PCR product. **B**, Percent resection is calculated from PCR values for undigested and digested DNA at 3.3- and 12.6-kb away from an HO break at the *MAT* locus as described in Materials and Methods. Percent resection in resection-deficient *htz1*Δ mutant cells is shown as a control.
(TIF)

S9 Fig *hst3*Δ *hst4*Δ cells are not defective in the strand invasion step of homologous recombination. The enrichment of Rad51 flanking the recipient (*MAT*) and donor (*HML*) sequences is determined by quantitative PCR. The locations of the HO break and the primers are shown. The results are the mean values from three independent experiments ± s.d.
(TIF)

S10 Fig *hst3*Δ *hst4*Δ cells are moderately defective in non-homologous tail (3’ flap) removal. A plasmid-based non-homologous tail removal assay was used to determine the efficiency of flap removal. **A**, pFP122 contains two intact *Lac*Z genes, one of which contains an HO cut site whereas the other site is mutated. pFP120 contains one intact *Lac*Z gene with an intact HO cut site and the second *Lac*Z gene with an HO cut site and 308- and 610-bp flanking sequence deleted. The plasmids also carry *CEN4* and *URA3* as a marker. **B**, The ratio of retention rate (Ura^+^ events) between pFP122 and pFP120 was used to calculate the efficiency of 3’ flap removal. Plotted are the mean values of three independent experiments ± s.d.
(TIF)

S11 Fig BIR synthesis was determined by semi-quantitative PCR in cells depleted of Hst3, or with mock depletion. **A**, PCR was performed using upstream PCR primer (F2) annealing to the *NPR2* gene and downstream primer (R3) annealing to the carboxy terminus of *CAN1* gene. **B**, Representative DNA agarose gel images of semi-quantitative PCR. **C**, Quantification of the gel images. Plotted are the means of percent repair products at indicated time points post-HO expression from two independent experiments ± s.d.
(TIF)

S12 Fig Pol3 recruitment at donor template is deficient in *hst3*Δ *hst4*Δ cells during BIR. Cells with indicated genotypes of disomic BIR strains were cultured in preinduction YEP-glycerol media, induced HO for 4 h, and harvested for chromatin IP analysis to detect Pol1-3HA, Pol2-3HA, Pol3-13Myc and Pif1-3HA enrichment at homologous template during BIR. Primers used in quantitative PCR were 0.4-kb downstream of *MAT*ATP-glycerol*TAF2* gene.
(TIF)

S13 Fig Hypothetical model of collapsed fork recovery in *mrc1* or *tof1* cells. In *mrc1Δ* or *tof1Δ* cells, replisome is uncoupled from DNA synthesis upon HU arrest, which cause 2–3 kb ssDNA (Katou et. al Nature 2003) that is subjected to occasional breakage. Followed by an arrival of a rescuing fork from opposite direction finishes DNA synthesis by filling in the remaining ssDNA gap and deposition of nucleosomes carrying acetylated H3K56 (orange circles) at intact DNA strand. Fork recovery then requires 2–3 kb gapped repair synthesis across DNA with acetylated H3K56.
(TIF)

S14 Fig The level of DNA breaks in *rad53*Δ, *mrc1*Δ, *pol32Δ mrc1Δ* and *hst3*Δ *hst4*Δ *mrc1*Δ after acute HU treatment. **A**, The flowchart of acute HU treatment. **B**, PFGE analysis of chromosome samples harvested at different time points.
(TIF)

S1 Table Representative examples of chromosomal translocation breakpoints in *hst3 Δ hst4 Δ* cells. (TIF)

S2 Table List of yeast strains. (PDF)
