# Supplementary figures and images for: Hyper-Acetylation of Histone H3K56 Limits Break-Induced Replication by Inhibiting Extensive Repair Synthesis
Source: PLoS Genet. 2015 Feb 23;11(2):e1004990. doi: 10.1371/journal.pgen.1004990 (PMC4338291; doi:10.1371/journal.pgen.1004990)

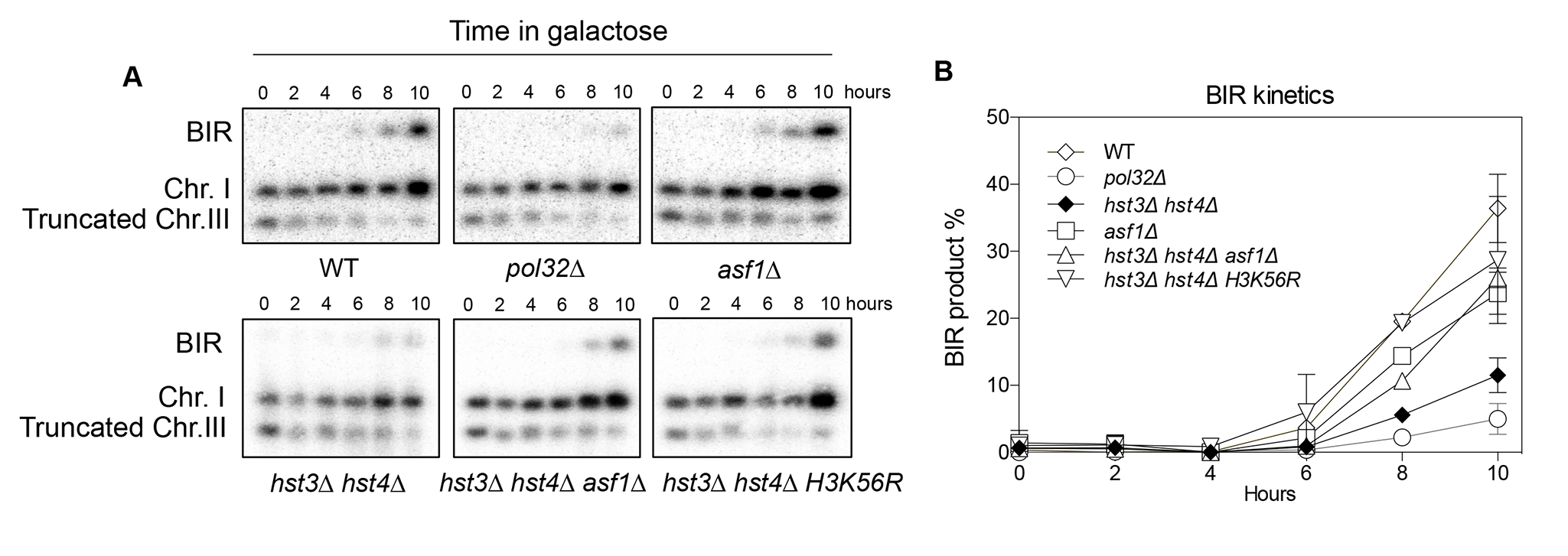

Supplement: S1 Fig — A, Southern blot analysis of BIR product formation. Chromosomes were separated by PFGE and a DNA probe specific for ADE1 was used to detect BIR products. B, Quantification of Southern blot results. (TIF) [file pgen.1004990.s001.tif]

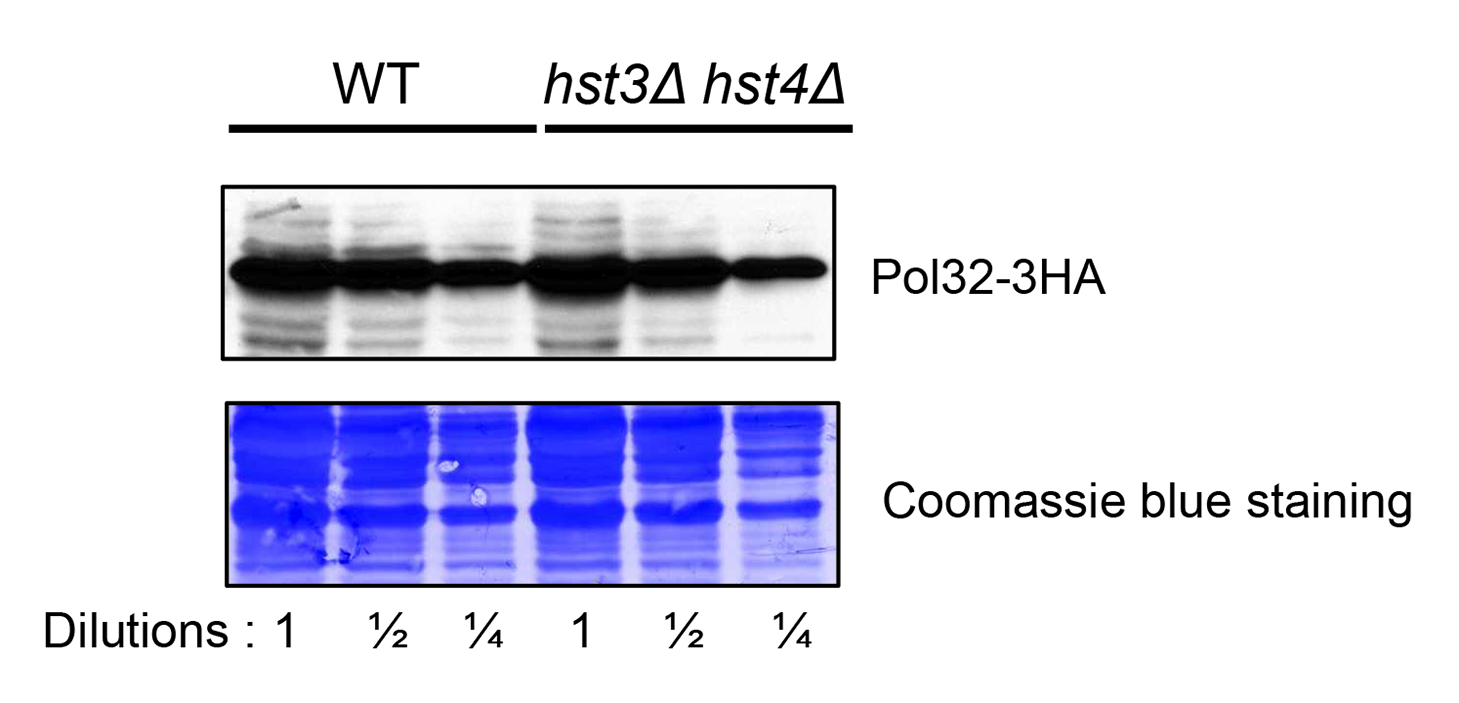

Supplement: S2 Fig — Cycling cells with Pol32-3xHA were harvested and Western blotting was performed to determine the level of Pol32 expression in hst3Δ hst4Δ cells. Samples were 1:2 serially diluted for better resolution. Coomassie blue stained PVDF membrane is shown as a loading control. (TIF) [file pgen.1004990.s002.tif]

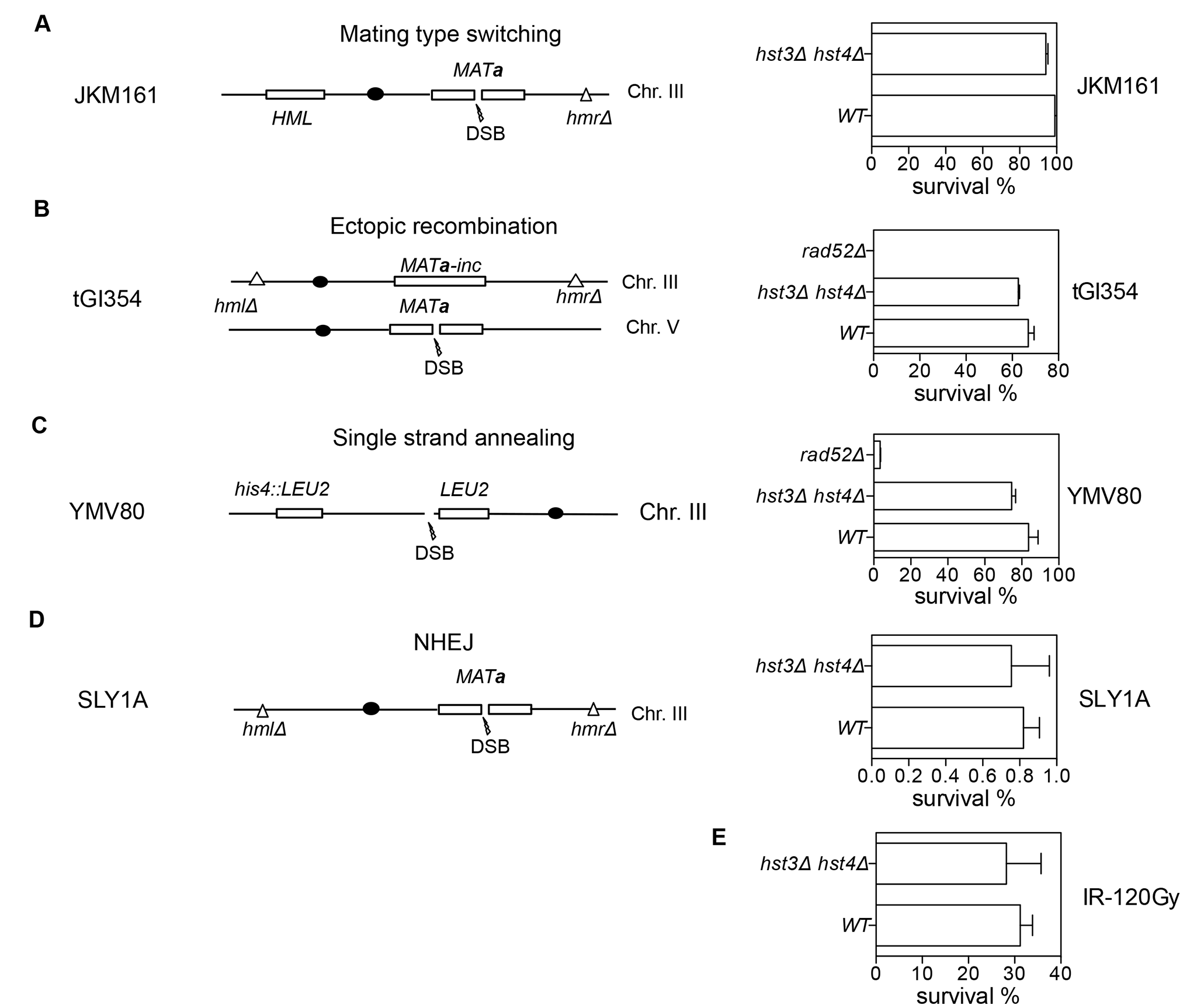

Supplement: S3 Fig — Left: Schematic diagram of genetic assays analyzing the integrity of mating-type switching (A), ectopic recombination (B), single strand annealing (C) and non-homologous end joining (D). Right: Efficiency of repair in hst3Δ hst4Δ as measured by viability following a DSB or 120 Gray ionizing radiation (IR) (E). Error bars represent s.d. (TIF) [file pgen.1004990.s003.tif]

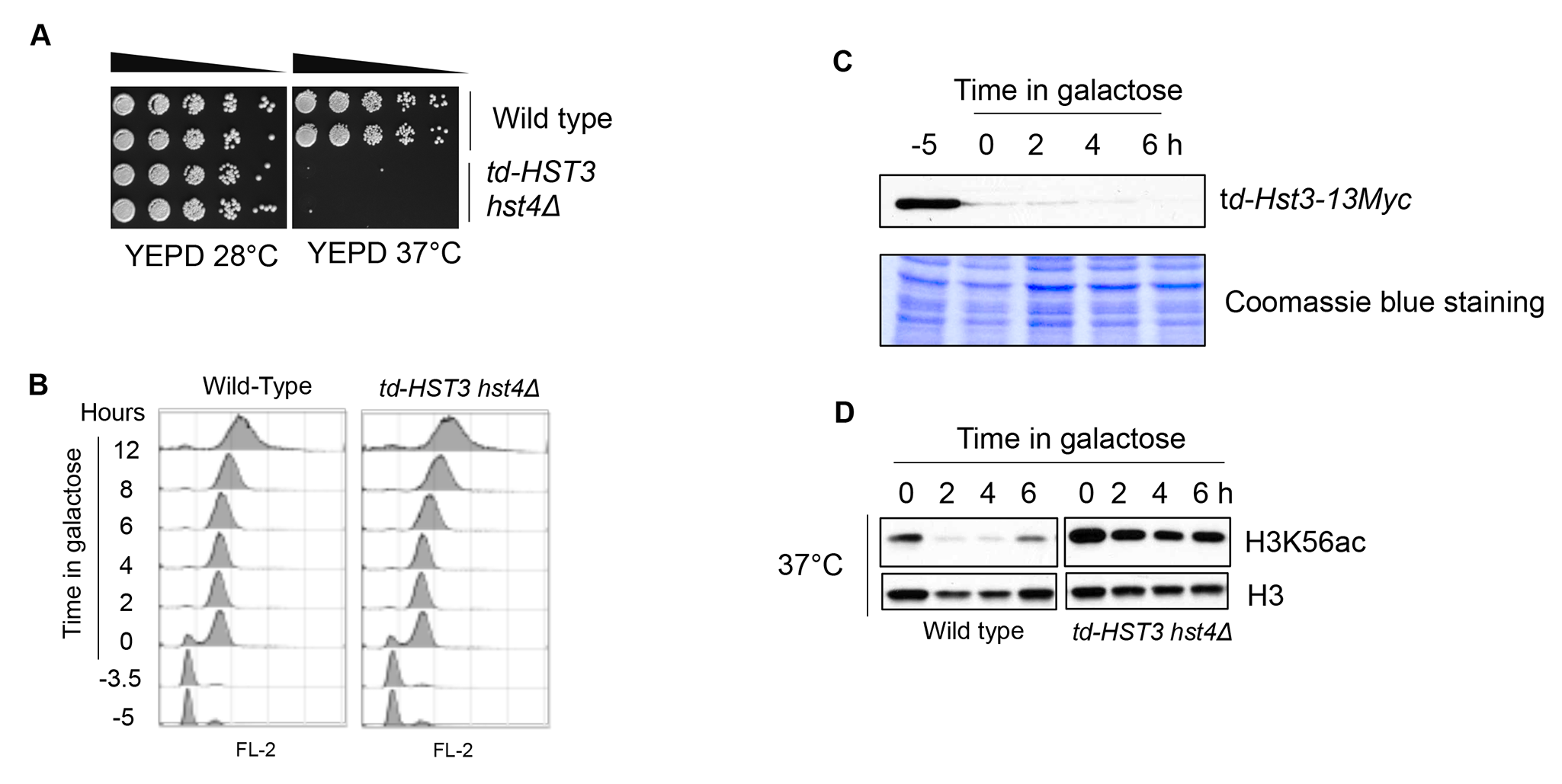

Supplement: S4 Fig — A, Serial dilutions of hst4Δ cells with or without degron fused (td)-Hst3 were spotted and cultured at 28°C or 37°C. Pictures were taken after 4 days. B, Cell cycle progression monitored by fluorescence activated cell sorting (FACS). C, The level of Hst3 expression in cells growing at non-permissive (37°C) temperature. Coomassie blue stained PVDF membrane is shown as a loading control. D, The level of H3K56 acetylation upon degron-induced depletion of Hst3. The level of H3 is shown as a loading control. (TIF) [file pgen.1004990.s004.tif]

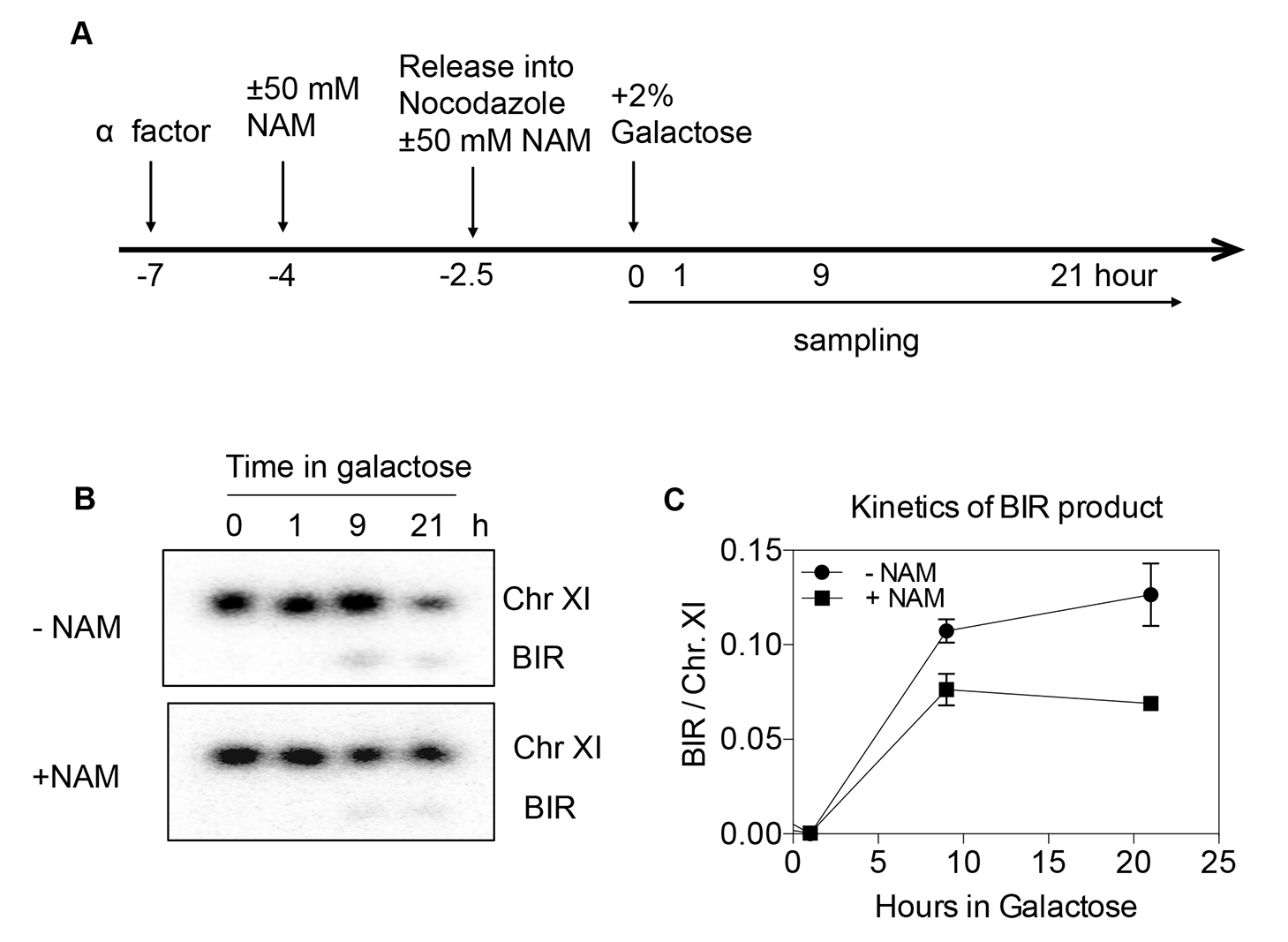

Supplement: S5 Fig — A, Flowchart showing experimental procedure. B, Southern blot analysis of BIR product formation in cells treated with 50 mM NAM. Chromosomes were separated by PFGE and a DNA probe specific for MCH2 was used to detect BIR products. C, Quantification of Southern blot results. (TIF) [file pgen.1004990.s005.tif]

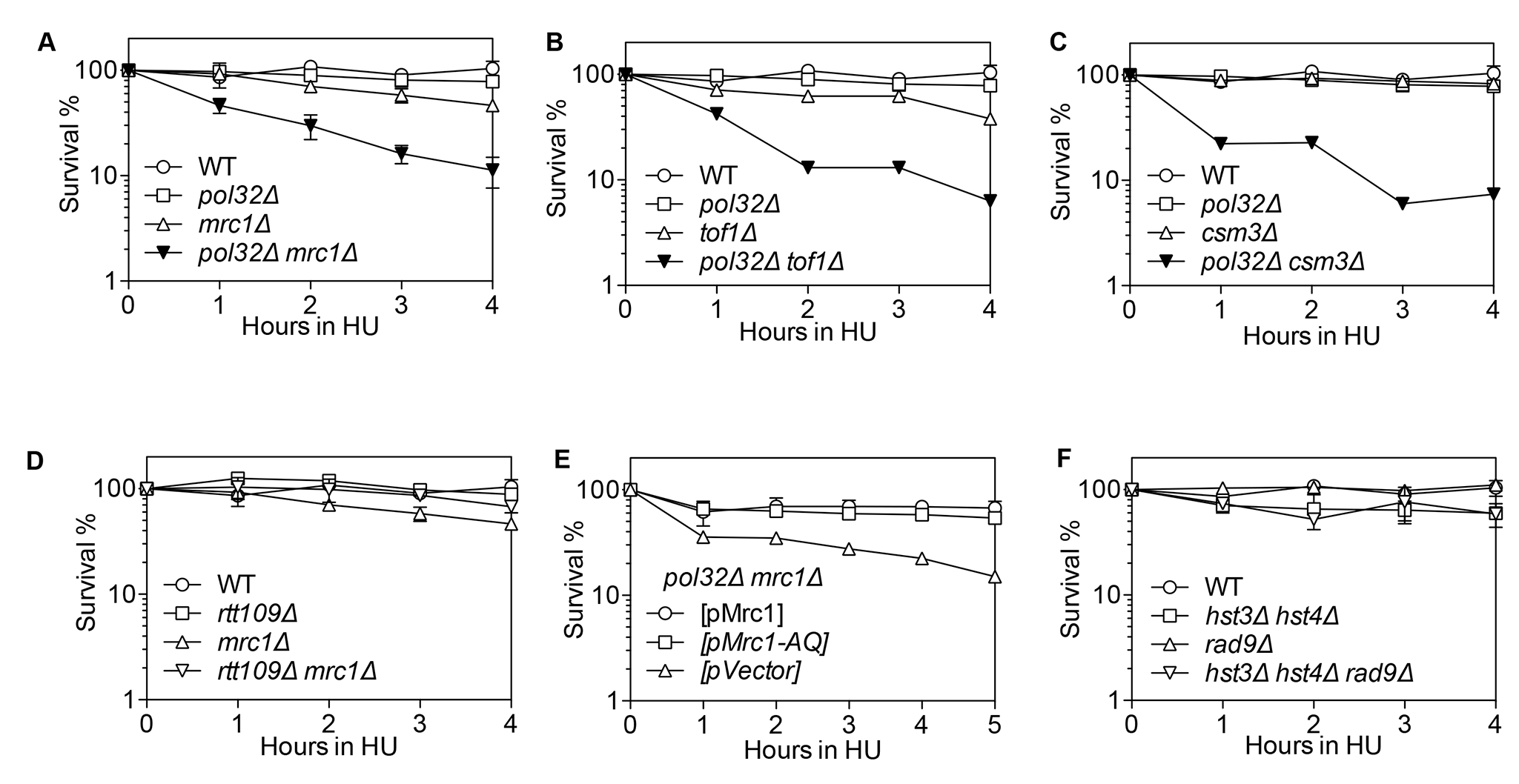

Supplement: S6 Fig — Percent survival after 150 mM HU for up to 5 h was determined in cells with indicated genotypes: A, pol32Δ, mrc1Δ, pol32Δ mrc1Δ; B, pol32Δ, tof1Δ and pol32Δ tof1Δ; C, pol32Δ, csm3Δ, and pol32Δ csm3Δ; D, rtt109Δ, mrc1Δ, and rtt109Δ mrc1Δ; E, pol32Δ mrc1Δ supplemented with the plasmids expressing Mrc1 or mrc1AQ, F, hst3Δ hst4Δ, rad9Δ and hst3Δ hst4Δ rad9Δ. Plotted are the mean values of three independent experiments ± s.d. (TIF) [file pgen.1004990.s006.tif]

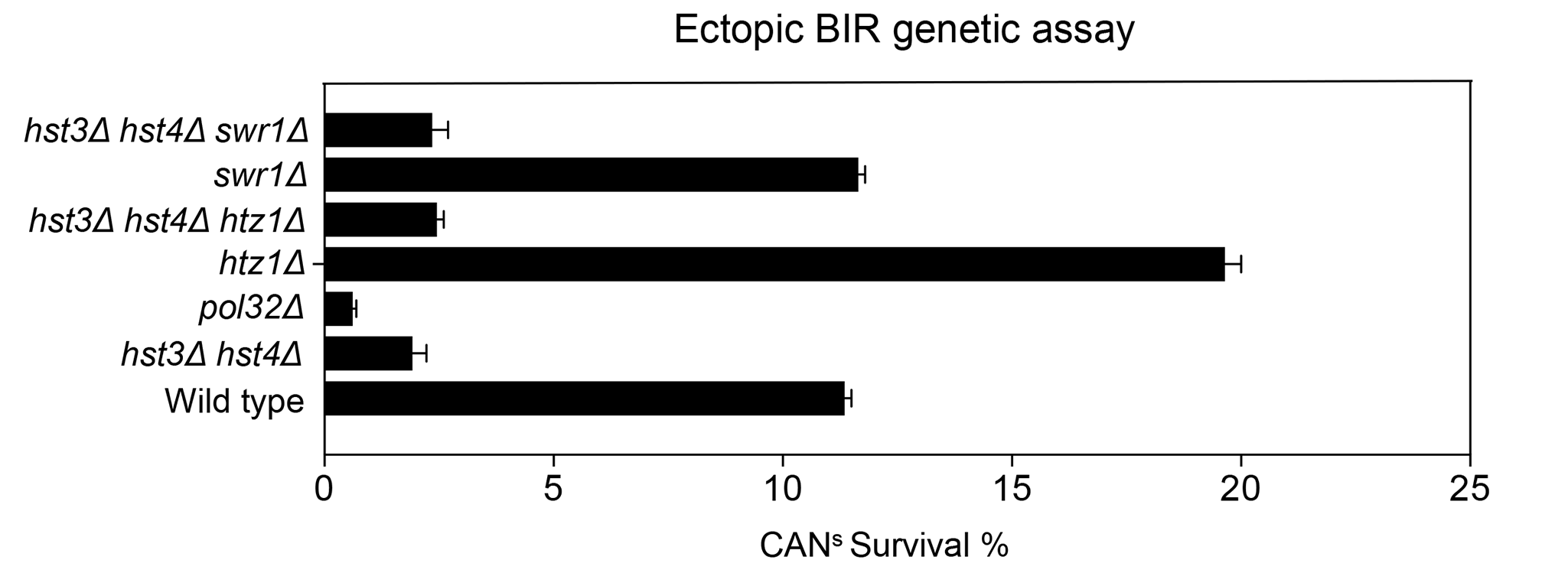

Supplement: S7 Fig — Efficiency of BIR in hst3Δ hst4Δ, hst3Δ hst4Δ htz1Δ, and hst3Δ hst4Δ swr1Δ cells, as measured by canavanine sensitive colony formation following a DSB using the ectopic BIR assay. (TIF) [file pgen.1004990.s007.tif]

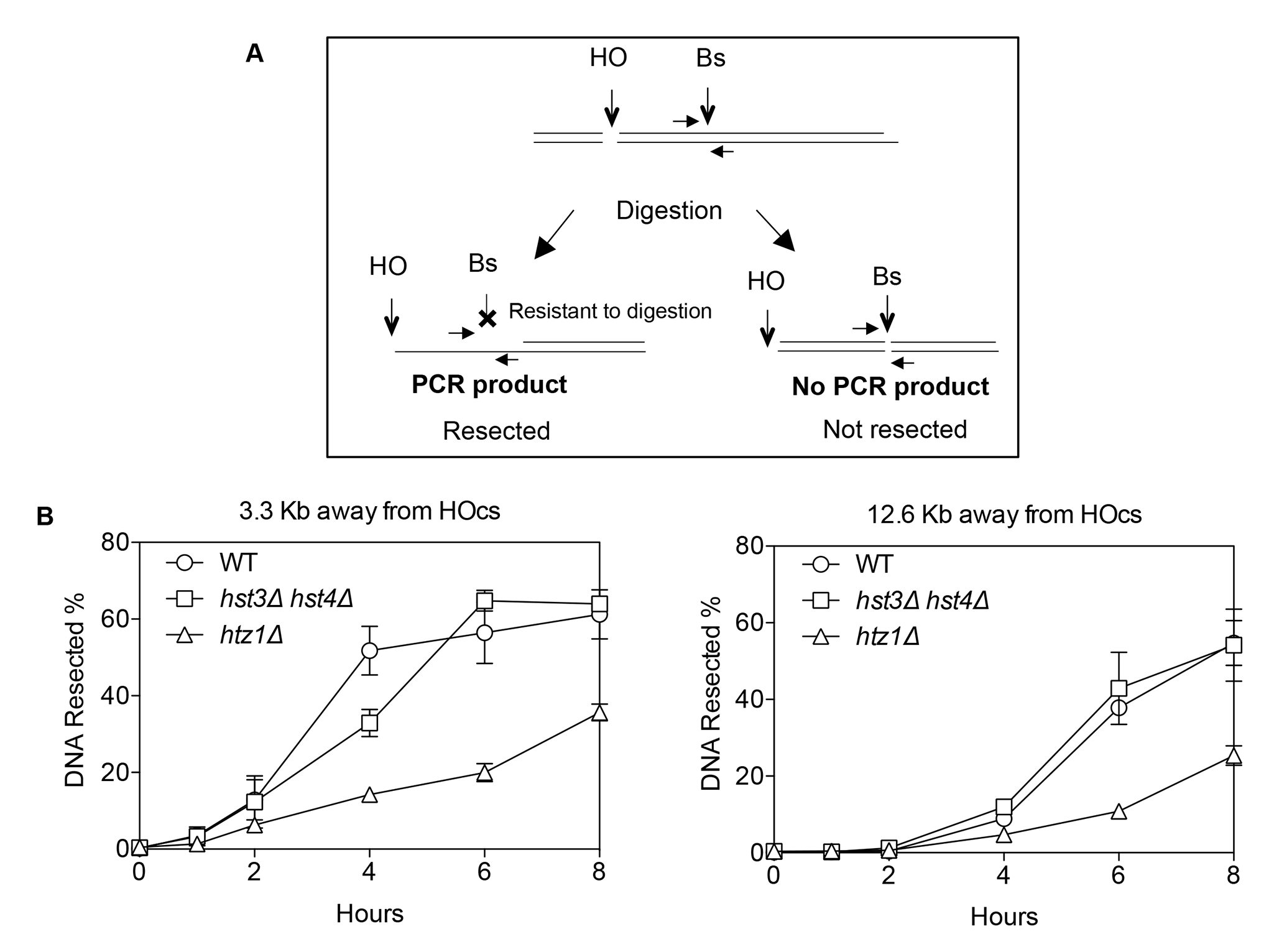

Supplement: S8 Fig — A, Schematic diagram illustrating the principle of determining DNA resection by quantitative PCR. When DNA is not resected (not resected), DNA can be digested by restriction enzyme (BsaJI, Bs). PCR primers surrounding the restriction enzyme site (grey arrows) cannot yield a PCR product. Upon resection to generate single stranded DNA, the restriction site is lost, and PCR primers surrounding the restriction site yield a PCR product. B, Percent resection is calculated from PCR values for undigested and digested DNA at 3.3- and 12.6-kb away from an HO break at the MAT locus as described in Materials and Methods. Percent resection in resection-deficient htz1Δ mutant cells is shown as a control. (TIF) [file pgen.1004990.s008.tif]

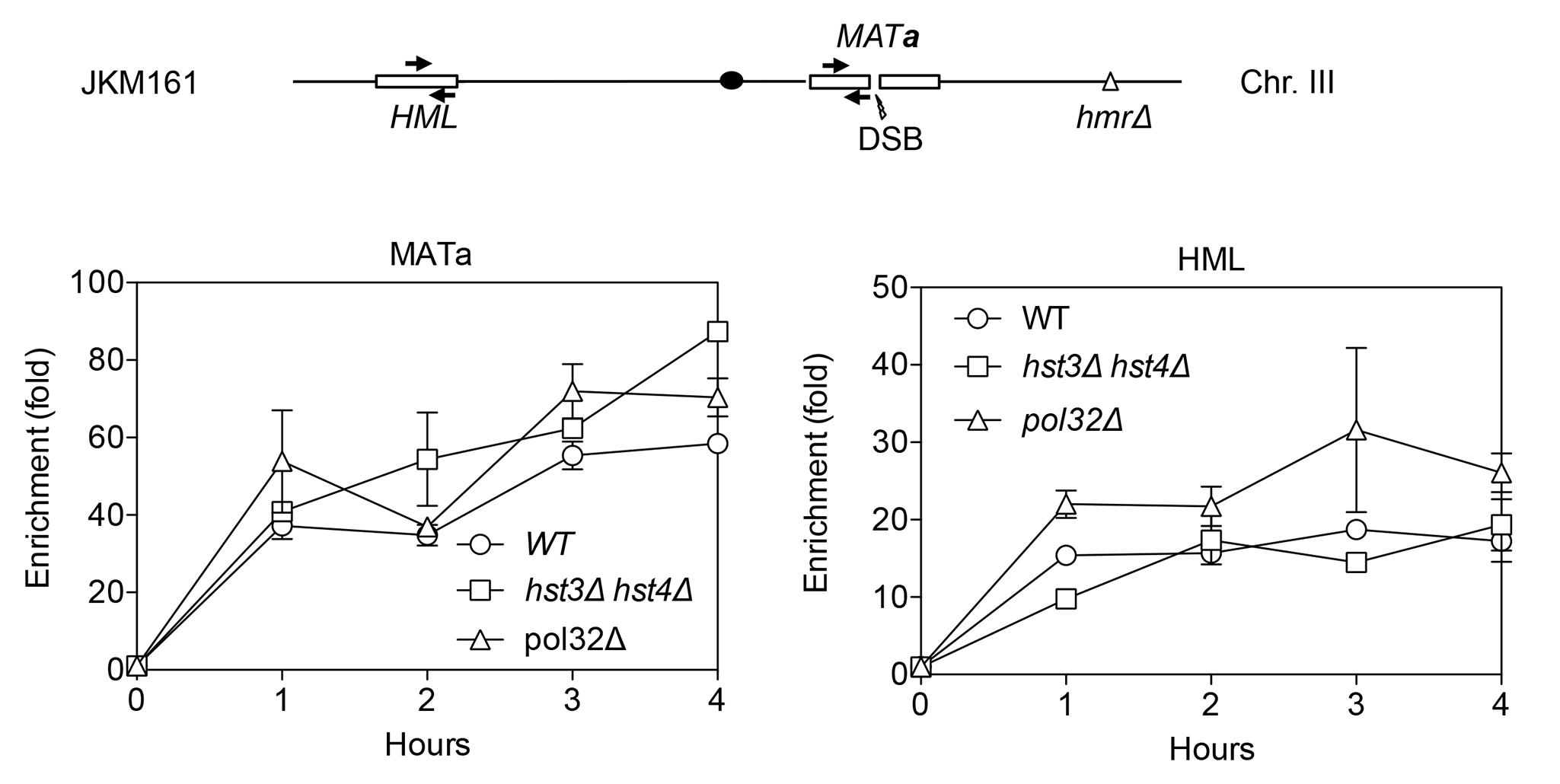

Supplement: S9 Fig — The enrichment of Rad51 flanking the recipient (MAT) and donor (HML) sequences is determined by quantitative PCR. The locations of the HO break and the primers are shown. The results are the mean values from three independent experiments ± s.d. (TIF) [file pgen.1004990.s009.tif]

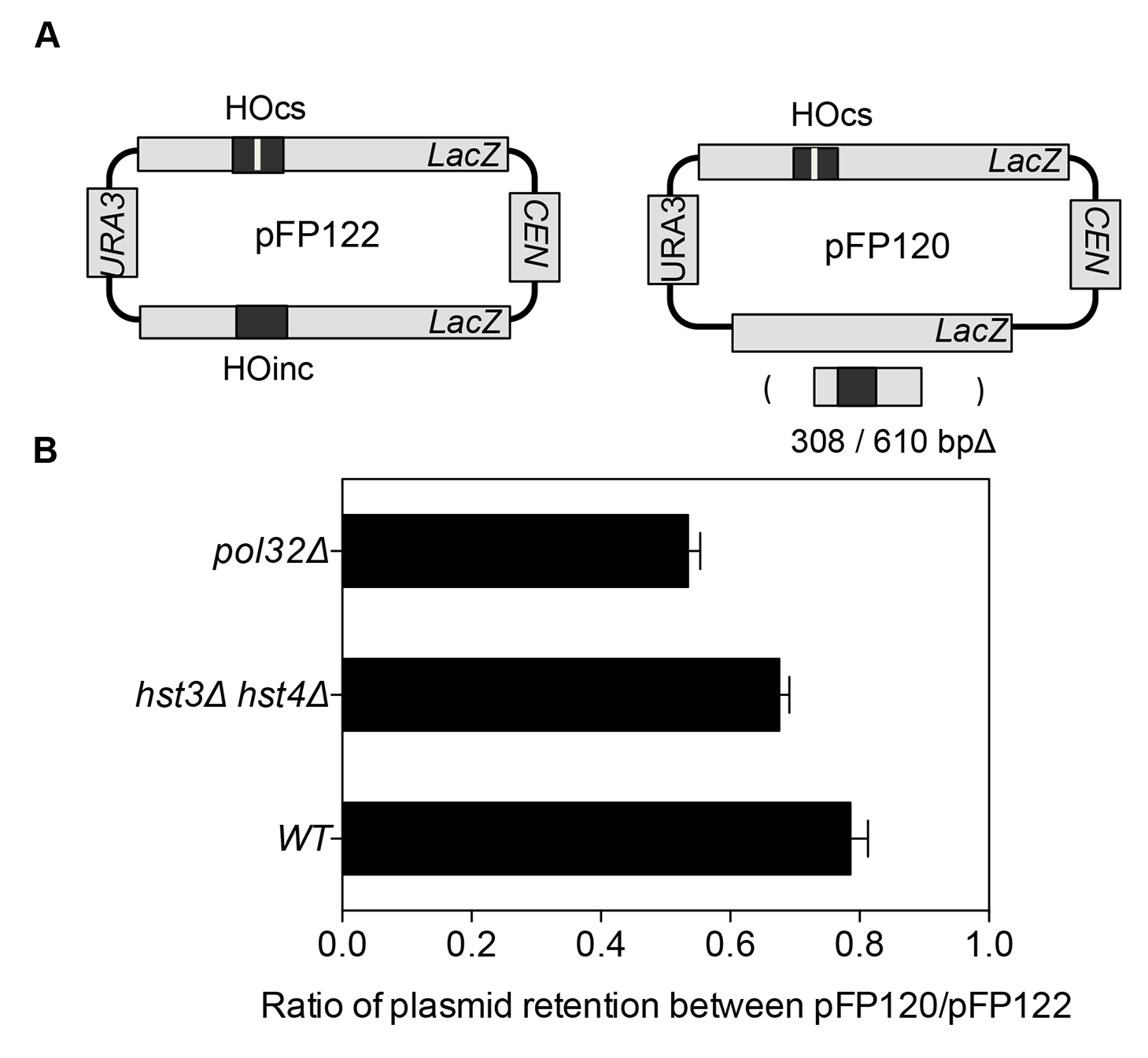

Supplement: S10 Fig — A plasmid-based non-homologous tail removal assay was used to determine the efficiency of flap removal. A, pFP122 contains two intact LacZ genes, one of which contains an HO cut site whereas the other site is mutated. pFP120 contains one intact LacZ gene with an intact HO cut site and the second LacZ gene with an HO cut site and 308- and 610-bp flanking sequence deleted. The plasmids also carry CEN4 and URA3 as a marker. B, The ratio of retention rate (Ura+ events) between pFP122 and pFP120 was used to calculate the efficiency of 3’ flap removal. Plotted are the mean values of three independent experiments ± s.d. (TIF) [file pgen.1004990.s010.tif]

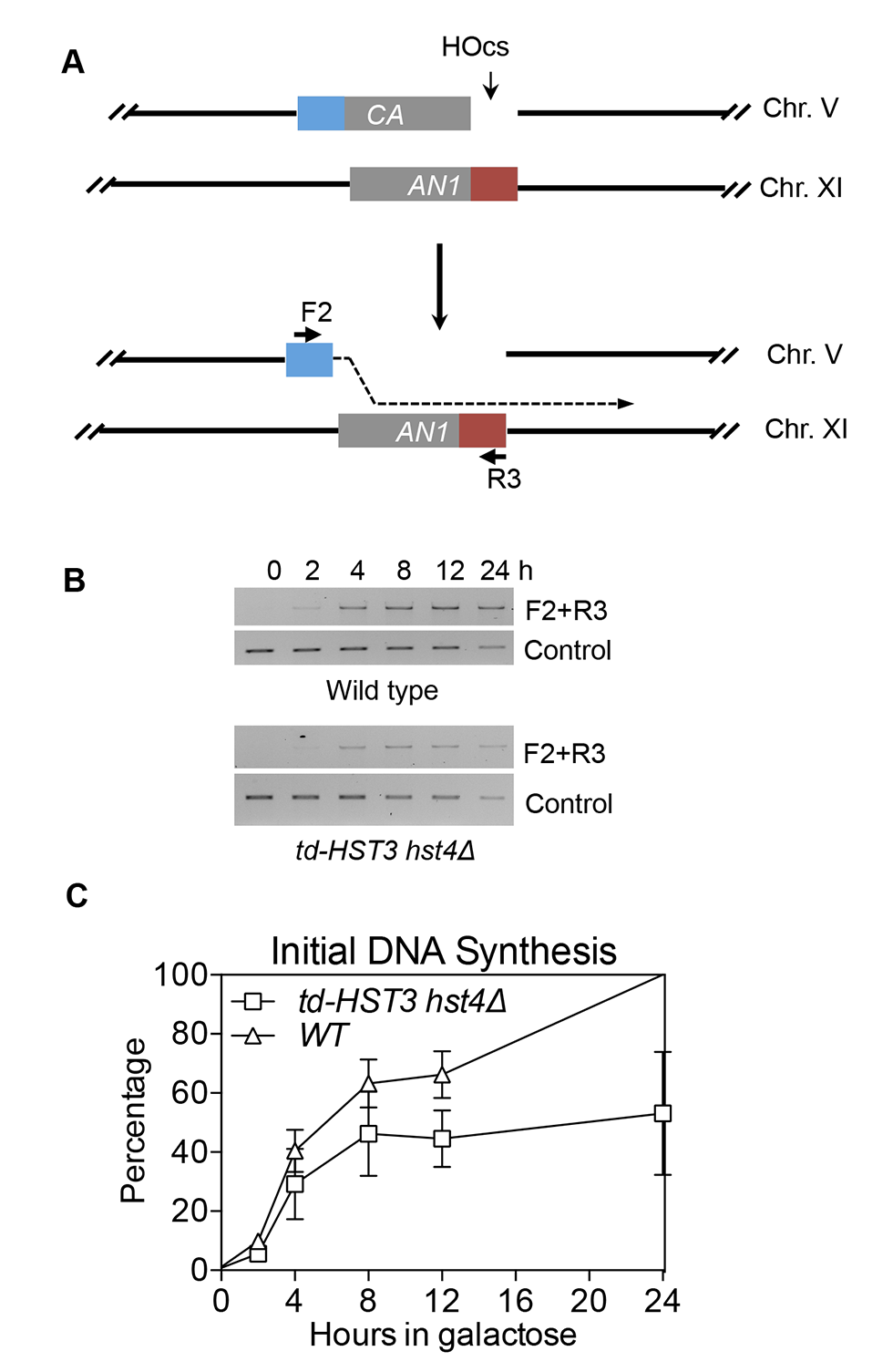

Supplement: S11 Fig — A, PCR was performed using upstream PCR primer (F2) annealing to the NPR2 gene and downstream primer (R3) annealing to the carboxy terminus of CAN1 gene. B, Representative DNA agarose gel images of semi-quantitative PCR. C, Quantification of the gel images. Plotted are the means of percent repair products at indicated time points post-HO expression from two independent experiments ± s.d. (TIF) [file pgen.1004990.s011.tif]

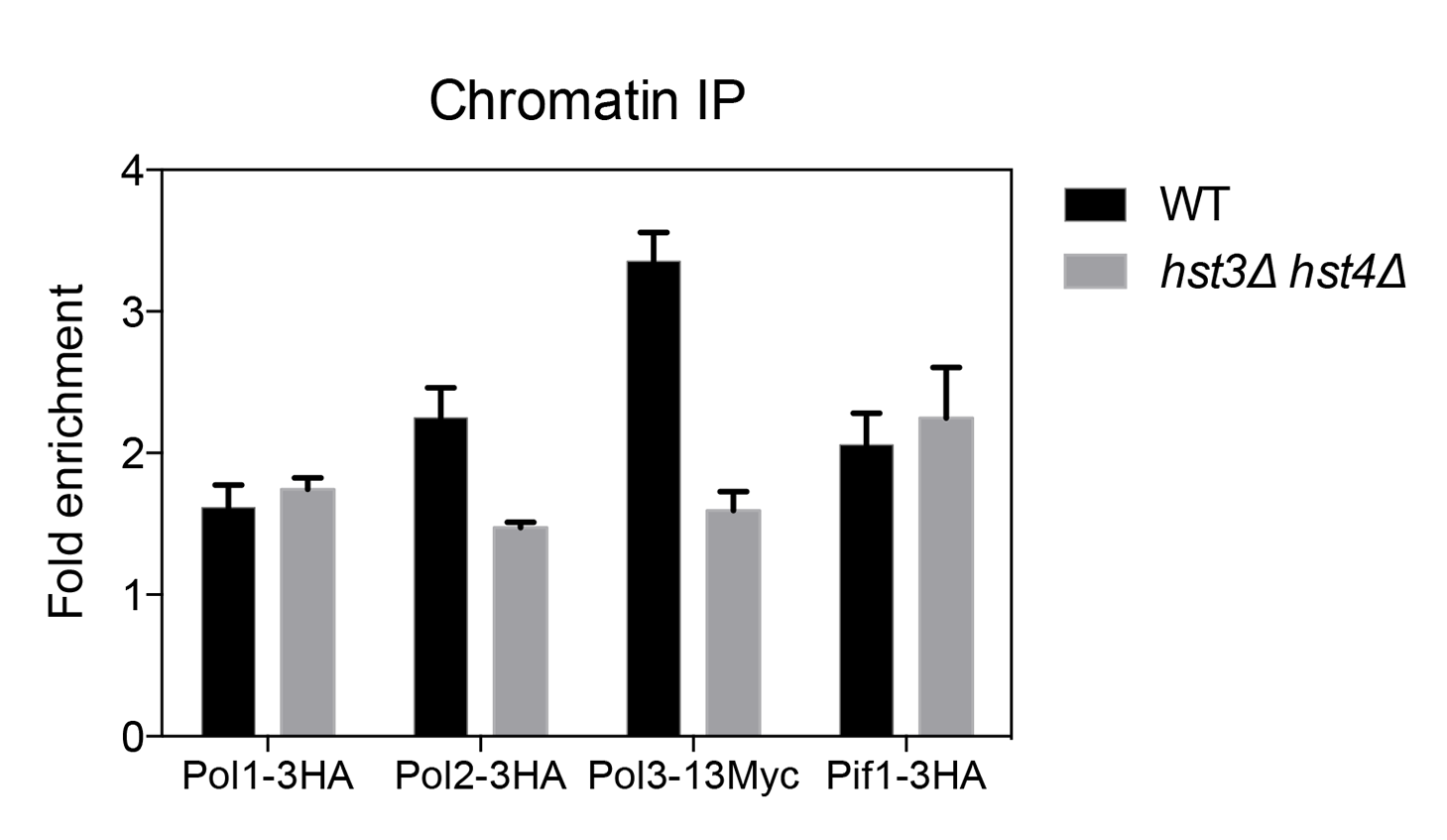

Supplement: S12 Fig — Cells with indicated genotypes of disomic BIR strains were cultured in preinduction YEP-glycerol media, induced HO for 4 h, and harvested for chromatin IP analysis to detect Pol1-3HA, Pol2-3HA, Pol3-13Myc and Pif1-3HA enrichment at homologous template during BIR. Primers used in quantitative PCR were 0.4-kb downstream of MATATP-glycerolTAF2 gene. (TIF) [file pgen.1004990.s012.tif]

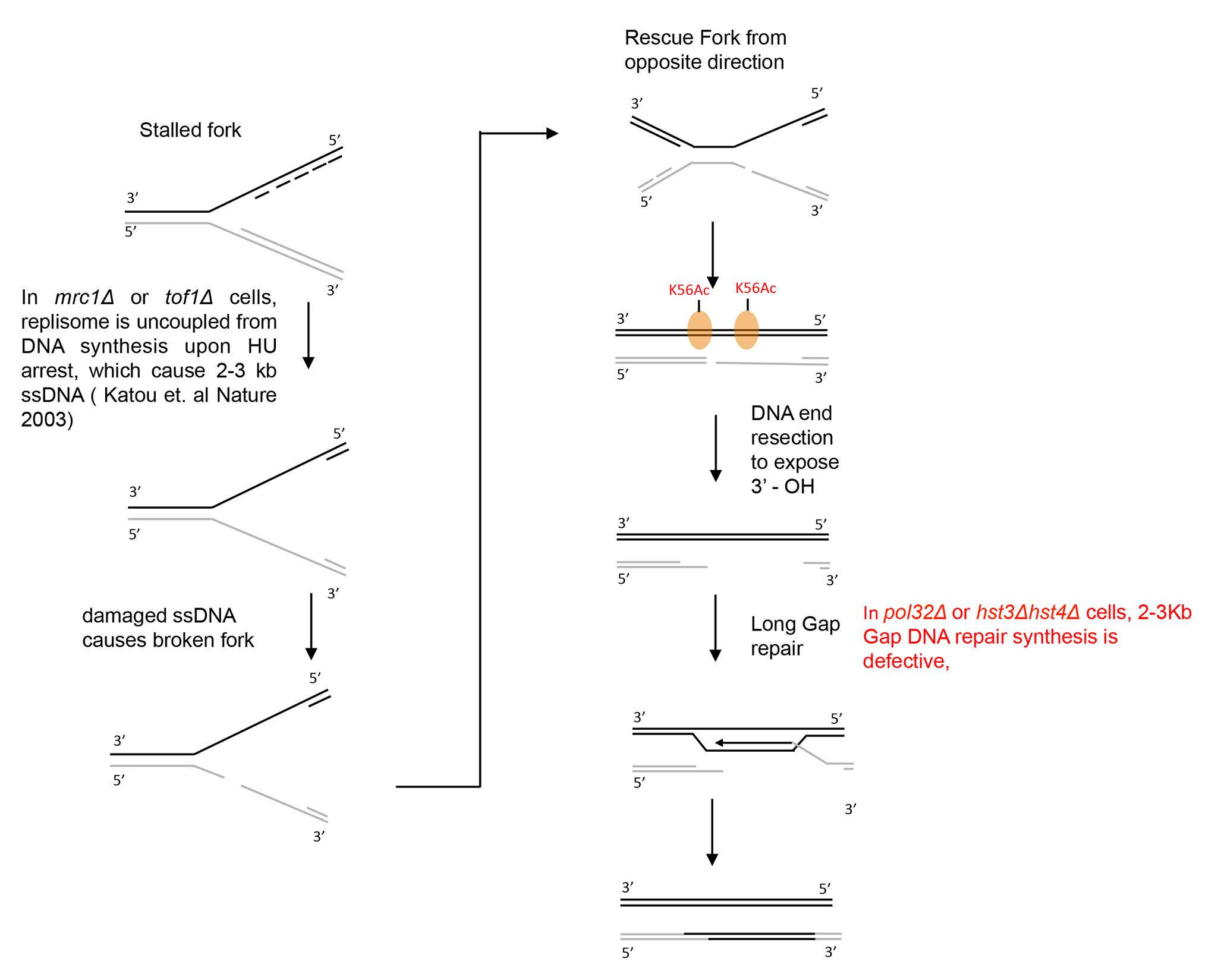

Supplement: S13 Fig — In mrc1Δ or tof1Δ cells, replisome is uncoupled from DNA synthesis upon HU arrest, which cause 2–3 kb ssDNA (Katou et. al Nature 2003) that is subjected to occasional breakage. Followed by an arrival of a rescuing fork from opposite direction finishes DNA synthesis by filling in the remaining ssDNA gap and deposition of nucleosomes carrying acetylated H3K56 (orange circles) at intact DNA strand. Fork recovery then requires 2–3 kb gapped repair synthesis across DNA with acetylated H3K56. (TIF) [file pgen.1004990.s013.tif]

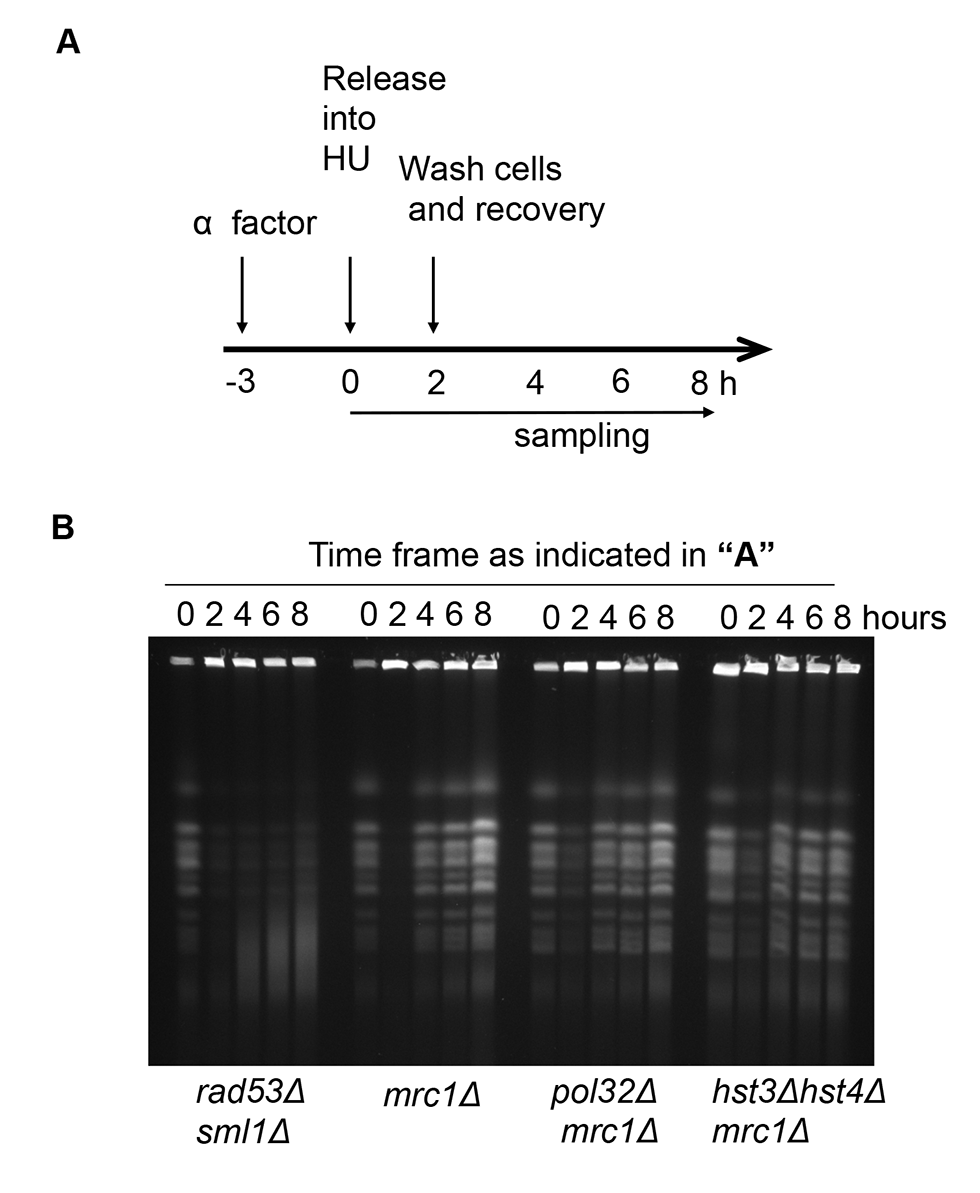

Supplement: S14 Fig — A, The flowchart of acute HU treatment. B, PFGE analysis of chromosome samples harvested at different time points. (TIF) [file pgen.1004990.s014.tif]

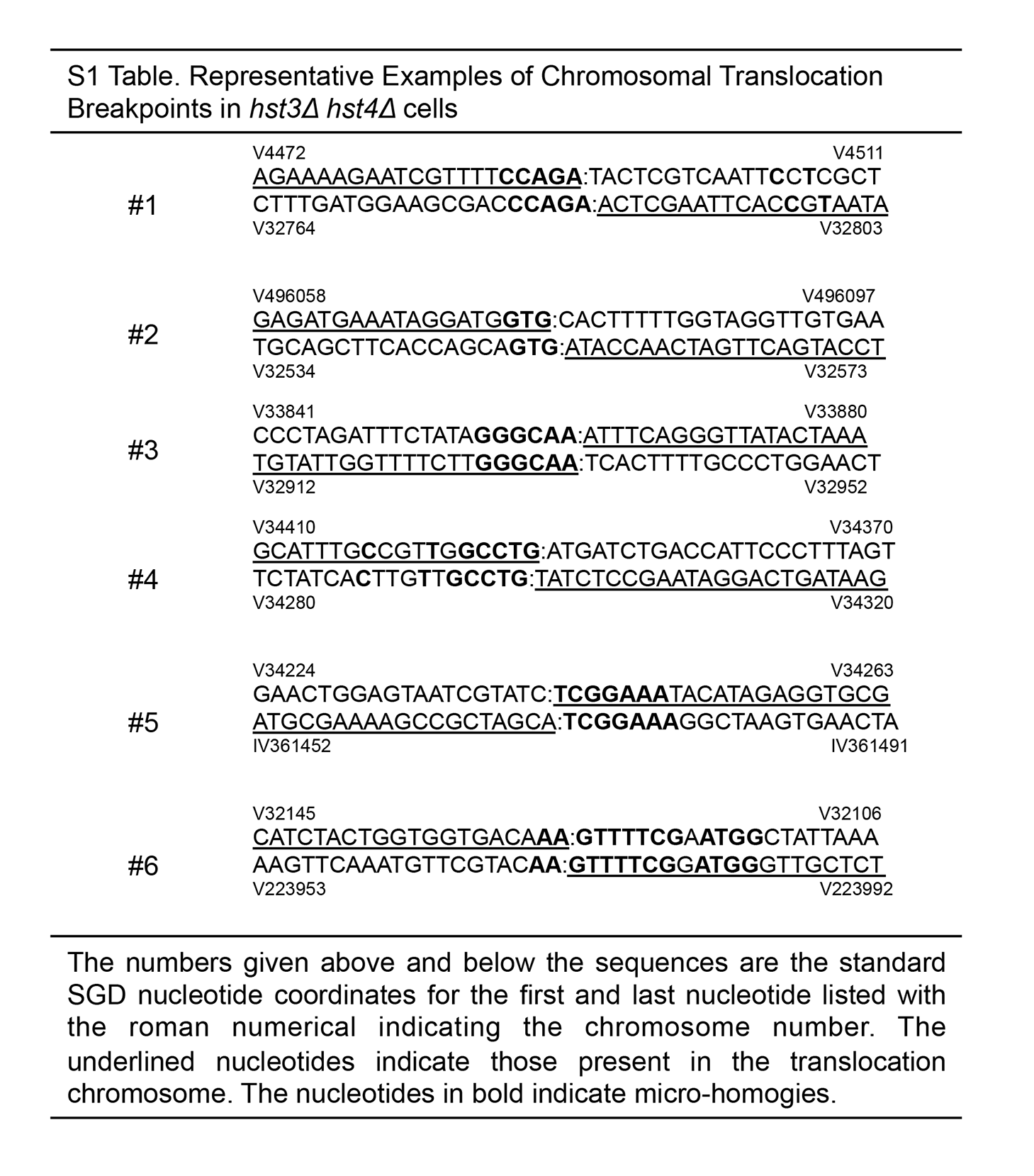

Supplement: S1 Table — (TIF) [file pgen.1004990.s015.tif]
