# Supplementary material for: Hyper-Acetylation of Histone H3K56 Limits Break-Induced Replication by Inhibiting Extensive Repair Synthesis
Source: PLoS Genet. 2015 Feb 23;11(2):e1004990. doi: 10.1371/journal.pgen.1004990 (PMC4338291; doi:10.1371/journal.pgen.1004990)
Supplement: S2 Table — (PDF) [file pgen.1004990.s016.pdf]

S2 Table List of yeast strains

Strain name	Genotype	Reference
AM1003	*MAT**a**-LEU2*-tel/*MAT*α*-inc ade1 met13 ura3 leu2-3,112/leu2 thr4 lys5 hml*Δ*::ADE1/hml*Δ*::ADE3 hmr*Δ*::HYG ade3::GAL-HO* FS2Δ*::NAT/*FS2	Deem et al. 2008 [1]
CY308	AM1003 *hst3Δ::KanMX*	This study
CY1180	AM1003 *hst4Δ::URA3*	This study
CY1115	AM1003 *hst3Δ::KanMX hst4Δ::URA3*	This study
CY1118	AM1003 *pol32Δ::KanMX*	This study
CY1177	AM1003 *rtt109Δ::URA3*	This study
CY1175	AM1003 *asf1Δ::URA3*	This study
CY1227	AM1003 *hst3Δ::KanMX hst4Δ rtt109Δ::URA3*	This study
CY1230	AM1003 *hst3Δ::KanMX hst4Δ asf1Δ::URA3*	This study
CY1404	AM1003 *hht2K56Q::Ura3 hht1Δ::KanMX* (H3K56Q)	This study
CY1448	AM1003 *hht2K56R::Ura3 hht1Δ::KanMX (H3K56R)*	This study
CY1452	AM1003 *hst3Δ::Thr4 hst4Δ htt2K56R::URA3 hht1Δ::KanMX*	This study
CY2048	AM1003 *hst1Δ::KanMX*	This study
CY2050	AM1003 *hst2Δ::KanMX*	This study
CY2051	AM1003 *sir2Δ::URA3*	This study
CY1454	AM1003 *swr1Δ::URA3*	This study
CY1455	AM1003 *htz1Δ::KanMX*	This study
CY1531	AM1003 *nhp10Δ::KanMX*	This study
CY1535	AM1003 *arp8Δ::KanMX*	This study
CY1763	AM1003 *gcn5Δ::KanMX*	This study
CY1765	AM1003 *hda1Δ::KanMX*	This study
CY1757	AM1003 *dot1Δ:: KanMX*	This study
CY1501	AM1003 *rad9Δ:: KanMX*	This study
CY1807	AM1003 *ctf4Δ:: KanMX*	This study
CY1810	AM1003 *hst3Δ::Thr4 hst4Δ ctf4Δ::KanMX*	This study
CY1834	AM1003 *mms22Δ::KanMX*	This study
CY1835	AM1003 *hst3Δ::Thr4 hst4Δ mms22Δ::KanMX*	This study
CY1805	AM1003 *hst3Δ::Thr4 hst4Δ rad17Δ::KanMX*	This study
CY2408	AM1003 *Pif1-3HA::KanMX*	This study
CY2413	AM1003 *Pol2-3HA::KanMX*	This study
CY2436	AM1003 *Pol3-13Myc:: Trp1*	Ira.G.
CY2470	AM1003 *Pol3-13Myc:: Trp1 hst3Δ::Thr4 hst4Δ::URA3*	This study
CY2467	AM1003 *Pif1-3HA:: KanMX hst3Δ::Thr4 hst4Δ::URA3*	This study
CY2419	AM1003 *Pol2-3HA::KanMX hst3Δ::Thr4 hst4Δ*	This study
CY1868	AM1003 *Pol1-3HA::KanMX*	This study
CY1870	AM1003 *Pol1-3HA::KanMX hst3Δ::Thr4 hst4Δ*	This study
AM1153	AM1003 Ura3 (For study initial DNA synthesis)	Deem et al. 2008[1]
CY1421	AM1153 *pol32Δ::KanMX*	This study
CY1437	AM1153 *hst3Δ::THR4 hst4Δ::KanMX*	This study
JRL346	*ho*Δ *mat::hisG hml*Δ*::hisG hmr*Δ*::ADE3 ura3*Δ*851 trp1*Δ*63 leu2*Δ*::KAN can1,1-1446::HOcs::HPH ykl215c::hisG-can1*Δ*289 ade3::GAL10::HO*	Lydread et al. 2010[2]

CY1902	JRL346 *pol32Δ::KanMX*	Haber JE
CY1927	JRL346 *hst3Δ::LEU2 hst4Δ::URA3*	This study
CY2778	JRL346 *td-HST3-13MYC::KanMX hst4Δ::LEU2*	This study
CY2057	JRL346 *htz1Δ::KanMX*	This study
CY2067	JRL346 *hst3Δ::LEU2 hst4Δ::URA3 htz1Δ::KanMX*	This study
CY2801	JRL346 *swr1Δ::KanMX*	This study
CY2803	JRL346 *hst3Δ::LEU2 hst4Δ::URA3 swr1Δ::KanMX*	This study
SLY1A	MATA *ade1 leu2-3,112 lys5 trp1*::hisG *ura3-52hml*::ADE1 *hmr*::ADE1 *ade3*::GAL::HO	Lee et al. 1998[3]
SLY2563	SLY1A *hst3Δ::KanMX hst4Δ::LEU2*	This study
CY420	*SLY1A mrc1Δ::HphMX*	This study
CY421	SLY1A *hst3Δ::KanMX hst4Δ::LEU2 mrc1Δ::HphMX*	This study
CY310	SLY1A *rad9Δ::HphMX*	This study
CY314	SLY1A *hst3Δ::KanMX hst4Δ::LEU2 rad9Δ::HphMX*	This study
CY1143	SLY1A *pol32Δ::KanMX*	This study
CY1245	SLY1A *mrc1Δ::HphMX pol32Δ::KanMX*	This study
CY1311	SLY1A *asf1Δ::KanMX*	This study
CY1313	SLY1A *rtt109Δ::KanMX*	This study
CY1317	SLY1A *mrc1Δ::HphMX asf1Δ::URA3*	This study
CY1319	SLY1A *mrc1Δ::HphMX rtt109Δ::URA3*	This study
CY1779	SLY1A *hst3ΔNatMX hst4Δ::LEU2 asf1Δ::KanMX mrc1Δ::HphMX*	This study
CY1650	SLY1A *hst3Δ::KanMX hst4::LEU2 mrc1Δ::HphMX [pMrc1-Ura3]*	This study
CY1653	SLY1A *hst3Δ::KanMX hst4::LEU2 mrc1Δ::HphMX [pMrc1-AQ-Ura3]*	This study
CY1656	SLY1A *hst3Δ::KanMX hst4::LEU2 mrc1Δ::HphMX [pRS316]*	This study
CY1669	SLY1A *pol32Δ::KanMX mrc1Δ::HphMX [pMrc1-Ura3]*	This study
CY1672	SLY1A *pol32Δ::KanMX mrc1Δ::HphMX [pMrc1-AQ-Ura3]*	This study
CY1675	SLY1A *pol32Δ::KanMX mrc1Δ::HphMX [pRS316]*	This study
CY1712	SLY1A *sml1Δ::HphMX rad53Δ::KanMx*	This study
CY1715	SLY1A *hst3Δ::NAT hst4Δ::LEU2 sml1Δ::HpHMX rad53Δ::KanMx*	This study
CY1340	SLY1A *tof1Δ::KanMX*	This study
CY1342	SLY1A *tof1Δ::KanMX pol32Δ::Ura3*	This study
CY1373	SLY1A *csm3Δ::KanMX*	This study
CY1377	SLY1A *csm3Δ::KanMX pol32Δ::Ura3*	This study
CY1759	SLY1A *POL32-3HA::KanMX*	This study
CY1761	SLY1A *hst3Δ::NAT hst4ΔLEU2 POL32-3HA::KanMX*	This study
CY2763	SLY1A *his3Δ*::ADE3 Rad52GFP::HIS3	This study
CY2761	SLY1A *hst3Δ::KanMX hst4Δ::LEU2 his3Δ*::ADE3 Rad52GFP::HIS3	This study
CY2769	SLY1A *hst3Δ::KanMX hst4Δ::LEU2 his3Δ*::ADE3 Rad52GFP::HIS3 *mrc1Δ::HphMX*	This study
14G10	BY4741 Rad52-GFP	Yeast GFP collection
CY1704	BY4741 Rad52-GFP *mrc1Δ*::HphMX	This study
CY1749	BY4741 Rad52-GFP *hst3Δ::KanMX hst4Δ::LEU2 mrc1Δ::HphMX*	This study
CY1750	BY4741 Rad52-GFP *pol32Δ::KanMX mrc1Δ::HphMX*	This study
CY1701	BY4741 Rad52-GFP *asf1::KanMX mrc1HphMX*	This study

CY2746	BY4741 Rad52-GFP *hst3Δ::KanMX hst4Δ::LEU2 asf1Δ::NAT mrc1Δ:: HphMX*	This study
YKJM1	MATa, *ura3-52, leu2Δ1, trp1Δ63, his3Δ200, lys2-Bgl, hom3-10, ade2Δ1, ade8, hxt13::*URA3	Myung et al. 2001[4]
CY300	YKJM1 *hst3Δ::KanMX hst4Δ::LEU2*	This study
CY1344	YKJM1 *asf1Δ::KanMX*	This study
CY1346	YKJM1 *rtt109Δ::KanMX*	This study
CY1347	YKJM1 *pol32Δ::KanMX*	This study
CY1528	YKJM1 *hst3Δ::KanMX hst4Δ::LEU2 asf1Δ::HIS3*	This study
JKM161	*Δho* HMLalpha MATa *Δhmr*::ADE1 *ade1–100 leu2–3,112 lys5 trp1::hisG ura3–52 ade3::*GAL-HO	Haber JE
CY1357	JKM161 *hst3Δ::KanMX hst4Δ::LEU2*	This study
CY1602	JKM161 *pol32Δ::KanMX*	This study
TGI354	*Δho Δhml::ADE1* MATa-inc *Δhmr*::ADE1 *ade1 leu2-3,112 lys5 trp1::hisG ura3-52 ade3*::GAL::HO *arg5,6*::GAL::MATa)	Prakash et al. 2009[5]
CY1023	TGI354 *hst3Δ::KanMX hst4Δ::LEU2*	This study
CY1840	TGI354 *rad52Δ::KanMX*	This study
CY1307	TGI354 *pol32Δ::KanMX*	This study
CY2536	TGI354-Gap	This study
CY2539	TGI354-Gap *hst3Δ::KanMX hst4Δ::LEU2*	This study
CY2542	TGI354-Gap *pol32Δ::KanMX*	This study
YMV80	*hmlΔ::*ADE1 *mataΔ::*hisG *hmrΔ::*ADE1 *leu2-cs ade3::*GAL::HO *ade1 lys5 ura3-52 leu2::his4*	Vaze et al. 2002[6]
CY320	YMV80 *hst3Δ::KanMX hst4Δ::URA3*	This study
CY1843	YMV80 *rad52Δ::KanMX*	This study

Reference

1. Deem A, Barker K, VanHulle K, Downing B, Vayl A, et al. (2008) Defective Break-Induced Replication Leads to Half-Crossovers in Saccharomyces cerevisiae. Genetics 179: 1845-1860.
2. Lydeard JR, Lipkin-Moore Z, Jain S, Eapen VV, Haber JE (2010) Sgs1 and exo1 redundantly inhibit break-induced replication and de novo telomere addition at broken chromosome ends. PLoS Genet 6: e1000973.
3. Lee SE, Moore JK, Holmes A, Umezu K, Kolodner RD, et al. (1998) Saccharomyces Ku70, mre11/rad50 and RPA proteins regulate adaptation to G2/M arrest after DNA damage. Cell 94: 399-409.
4. Myung K, Datta A, Kolodner RD (2001) Suppression of spontaneous chromosomal rearrangements by S phase checkpoint functions in Saccharomyces cerevisiae. Cell 104: 397-408.
5. Prakash R, Satory D, Dray E, Papusha A, Scheller J, et al. (2009) Yeast Mph1 helicase dissociates Rad51-made D-loops: implications for crossover control in mitotic recombination. Genes Dev 23: 67-79.
6. Vaze MB, Pellicioli A, Lee SE, Ira G, Liberi G, et al. (2002) Recovery from checkpoint-mediated arrest after repair of a double-strand break requires Srs2 helicase. Mol Cell 10: 373-385.
